# The crucial role of muscle glucocorticoid signaling in accelerating obesity and glucose intolerance via hyperinsulinemia

**DOI:** 10.1172/jci.insight.162382

**Published:** 2023-04-24

**Authors:** Hiroki Yamazaki, Masaaki Uehara, Noritada Yoshikawa, Akiko Kuribara-Souta, Motohisa Yamamoto, Yasuko Hirakawa, Yasuaki Kabe, Makoto Suematsu, Hirotoshi Tanaka

**Affiliations:** 1 Department of Biochemistry, Keio University School of Medicine, Tokyo, Japan.; 2 Department of Rheumatology and Allergy, IMSUT Hospital, Institute of Medical Science, The University of Tokyo, Tokyo, Japan.; 3 Center for Arthritis and Rheumatic Diseases, Kawasaki Municipal Hospital, Kanagawa, Japan.; 4 Department of Cell Processing and Transfusion, IMSUT Hospital, Institute of Medical Science, The University of Tokyo, Tokyo, Japan.; 5 Central Institute for Experimental Animals, Kawasaki City, Kanagawa, Japan.; 6 Department of Rheumatology, Kitasato University Kitasato Institute Hospital, Tokyo, Japan.

**Keywords:** Endocrinology, Muscle Biology, Insulin, Obesity

## Abstract

Metabolic crosstalk from skeletal muscle to multiple organs is important for maintaining homeostasis, and its dysregulation can lead to various diseases. Chronic glucocorticoid administration often induces muscle atrophy and metabolic disorders such as diabetes and central obesity; however, the detailed underlying mechanism remains unclear. We previously reported that the deletion of glucocorticoid receptor (GR) in skeletal muscle increases muscle mass and reduces fat mass through muscle-liver-fat communication under physiological conditions. In this study, we show that muscle GR signaling plays a crucial role in accelerating obesity through the induction of hyperinsulinemia. Fat accumulation in liver and adipose tissue, muscle atrophy, hyperglycemia, and hyperinsulinemia induced by chronic corticosterone (CORT) treatment improved in muscle-specific GR-knockout (GR-mKO) mice. Such CORT-induced fat accumulation was alleviated by suppressing insulin production (streptozotocin injection), indicating that hyperinsulinemia enhanced by muscle GR signaling promotes obesity. Strikingly, glucose intolerance and obesity in *ob/ob* mice without CORT treatment were also improved in GR-mKO mice, indicating that muscle GR signaling contributes to obesity-related metabolic changes, regardless of systemic glucocorticoid levels. Thus, this study provides insight for the treatment of obesity and diabetes by targeting muscle GR signaling.

## Graphical abstract

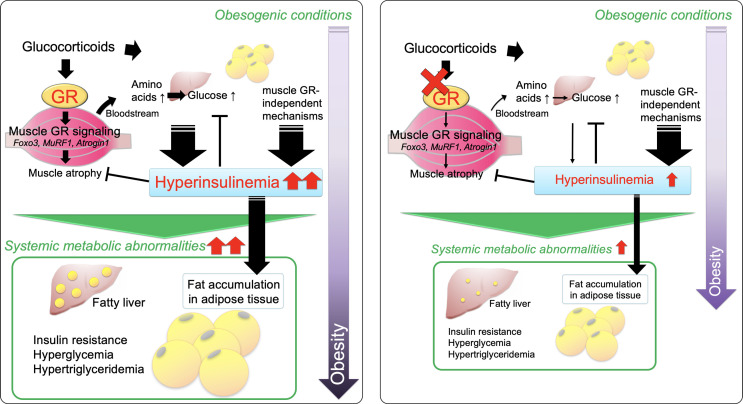

## Introduction

Complex organisms, including humans, maintain homeostasis by relying on the highly complicated and well-coordinated endocrine and nervous systems. Such systems control the peripheral organs, which interact with each other and also contribute to systemic homeostasis (1, 2).

Glucocorticoids are hormones secreted by the adrenal cortex under the regulation of the hypothalamic-pituitary-adrenal (HPA) axis and exert their effects through glucocorticoid receptors (GRs) present in various tissues, including the intestine, adipocytes, pancreas, liver, and skeletal muscle (3, 4). Skeletal muscle, not only playing a role in locomotor activity but also storing energy substrate mainly as protein (about 40% of body weight), supplies energy substrates under the regulation of the HPA axis during starvation via the glucose-alanine cycle (5, 6). In addition, we previously showed that muscle GR signaling regulates fat deposition in adipose tissues via a muscle-liver-fat signaling axis; increased muscle mass in muscle-specific GR-knockout (GR-mKO) mice reduces alanine excretion from skeletal muscle, and decreased alanine levels accelerate the production of fibroblast growth factor 21 (FGF21) in liver, leading to lipolysis in adipose tissue (7, 8). Thus, glucocorticoid-regulated interorgan communication involving muscle GR signaling plays various roles, at least in a physiological state.

Despite its physiological importance, excessive glucocorticoid levels can lead to metabolic abnormalities, including central obesity, fatty liver, glucose intolerance, and dyslipidemia, as clearly seen in Cushing’s syndrome or after long-term glucocorticoid administration (9). Even mild cortisol excess is related to central obesity, insulin resistance, and increased risk of cardiometabolic comorbidity (10–14). Mechanistically, it has been shown in vivo that chronic glucocorticoid administration promotes fat accumulation in adipose tissues and glucose intolerance via hepatocyte GR signaling (15), possibly in close association with the adipocyte GR signaling cascade, which regulates adipocyte differentiation and lipid metabolism (16, 17). Although glucocorticoid treatment induces muscle atrophy via muscle GRs (18), the contribution of muscle GR signaling to systemic fat accumulation and metabolic derangements such as glucose intolerance has not been well studied. Therefore, we first hypothesized that excessive glucocorticoid leads to its enhanced effect on skeletal muscle, which then alters systemic metabolism via interorgan communication and leads to systemic fat accumulation with glucose intolerance, in relation to glucocorticoid’s effects on liver and adipose tissues. We also hypothesized that muscle GR signaling under physiological glucocorticoid levels is associated with metabolic abnormalities in general obesity.

One of the most important metabolic hallmarks of obesity is hyperinsulinemia. Impaired responsiveness to insulin (insulin resistance) in peripheral tissues can lead to hyperglycemia and compensatory hyperinsulinemia, whereas hyperinsulinemia itself can promote insulin resistance through various mechanisms, including desensitization of insulin receptor signaling and enhancement of adipogenesis or lipogenesis (19, 20). Therefore, mild suppression of hyperinsulinemia without unfavorable metabolic changes is considered an interesting strategy to prevent or treat obesity (21).

In this study, we first show that fat accumulation in adipose tissue or liver, hyperinsulinemia, hyperglycemia, and hypertriglyceridemia induced by chronic corticosterone (CORT) treatment are mitigated in GR-mKO mice. We then demonstrate that hyperinsulinemia mediated by muscle GR is a primary factor inducing fat accumulation in adipose tissue (more minor effects in liver) and plays a counteractive role against muscle atrophy. Finally, we show that GR-mKO mice generated in an *ob/ob* background that were not treated with CORT also exhibit suppression of hyperinsulinemia followed by reduced fat accumulation, rendering insights into the role of hyperinsulinemia enhanced by muscle GR signaling in general obesity.

## Results

### Muscle GR signaling promotes muscle atrophy and systemic adiposity in CORT-induced obesity in mice.

**Figure 1:**
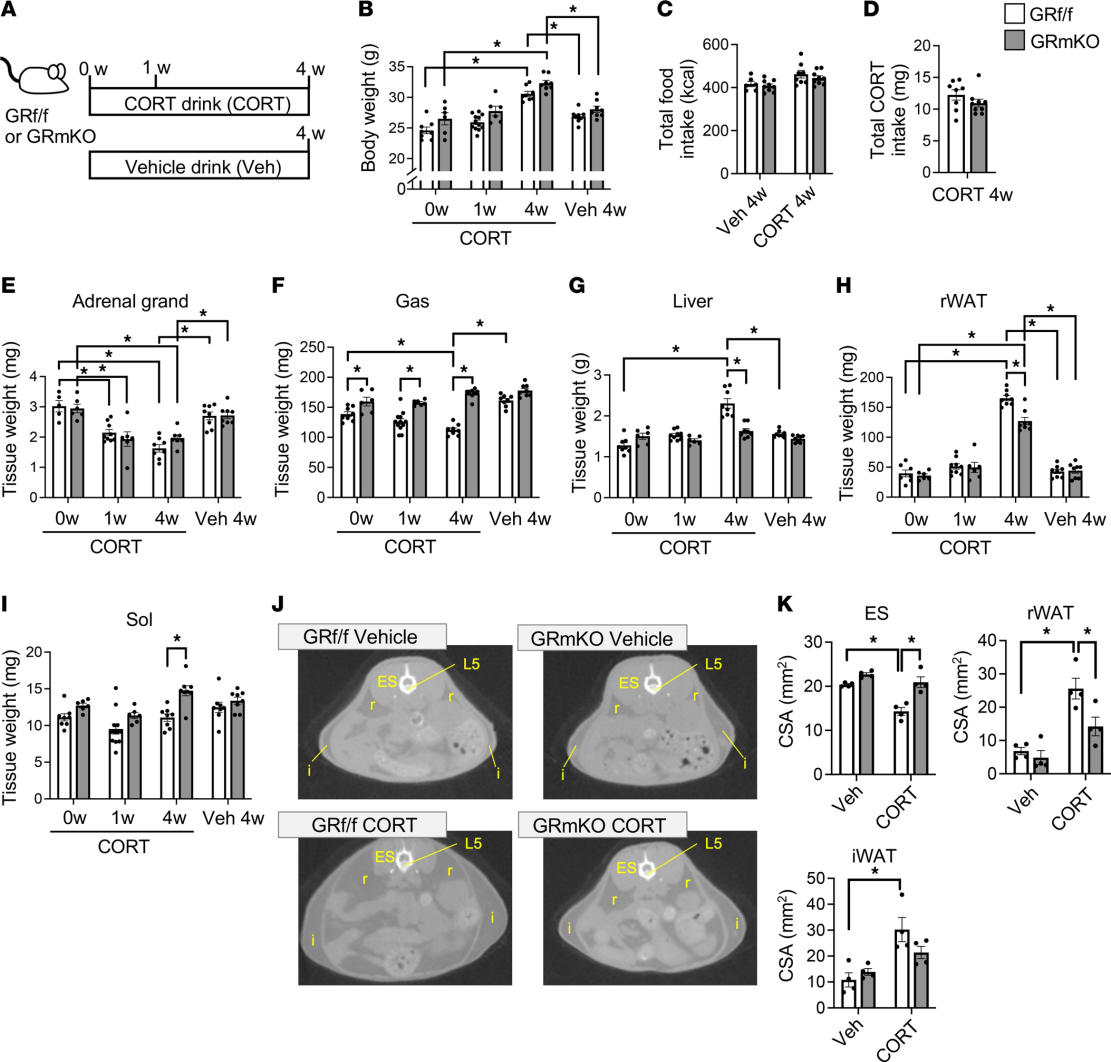
Skeletal muscle–specific GR-knockout (GR-mKO) mice are protected from body composition changes in an obese mouse model induced by chronic corticosterone (CORT) administration. (**A**) Experimental protocol. Eight-week-old GR^fl/fl^ and GR-mKO male mice were treated with vehicle or 100 μg/mL CORT solution for 0, 1, or 4 weeks. (**B**) Body weight changes. (**C**) Total food intake of mice treated with vehicle or CORT for 4 weeks. (**D**) Total CORT intake of CORT-treated mice for 4 weeks. (**E**–**I**) Tissue weights of adrenal gland (**E**), gastrocnemius muscle (Gas) (**F**), liver (**G**), retroperitoneal white adipose tissue (rWAT) (**H**), and soleus muscle (Sol) (**I**). Data are presented as mean ± SEM (*n* = 5–12). (**J**) Axial CT images at the L5 level for mice treated with vehicle or CORT for 4 weeks. L5, fifth lumbar vertebra; ES, erector spinae; r, rWAT; i, inguinal WAT (iWAT). (**K**) Cross-sectional areas (CSAs) of ES, rWAT, and iWAT measured using CT images at L5. Data presented as mean ± SEM (*n* = 4). In **D**, the statistical difference was evaluated using a 2-tailed Welch’s *t* test. For all others, 2-way ANOVA with Tukey-Kramer post hoc test was performed. Statistical differences were assessed in CORT at 0 weeks (0w) vs. CORT 1w, CORT 0w versus CORT 4w, and Veh 4w vs. CORT 4w for each genotype, and for GR^fl/fl^ versus GR-mKO under each condition. *P < 0.05.

To examine whether glucocorticoid action in skeletal muscle leads to systemic metabolic changes in the presence of excessive glucocorticoids, we first evaluated the significance of muscle GR signaling in body composition changes using a mouse model wherein chronically administered CORT drives systemic GR signaling, leading to obesity (22). CORT or vehicle solution in drinking water was administered to GR-floxed (GR^fl/fl^) and GR-mKO mice for 4 weeks (Figure 1A). CORT treatment did not impair muscle GR KO in GR-mKO mice (Supplemental Figure 1; supplemental material available online with this article; https://doi.org/10.1172/jci.insight.162382DS1). CORT treatment significantly increased the body weight of GR^fl/fl^ and GR-mKO mice (Figure 1B). The total food intake during the 4 weeks was not significantly different among the groups (Figure 1C). Ingested CORT and its bioavailability were comparable for GR^fl/fl^ and GR-mKO mice, as evidenced by the comparable total CORT intake (Figure 1D) and a progressive decrease in adrenal gland weight after CORT treatment in both genotypes (Figure 1E). Under these conditions, however, GR^fl/fl^ mice exhibited a CORT-induced decrease in the weight of gastrocnemius muscle and a CORT-induced increase in the weight of liver, neither of which were observed in GR-mKO mice (Figure 1, F and G). Additionally, the increase in the weight of retroperitoneal white adipose tissue (rWAT) was diminished in GR-mKO mice (Figure 1H). In the weight of soleus muscle, the change with CORT administration was minor (Figure 1I), suggesting that soleus muscle is less sensitive to CORT treatment than gastrocnemius muscle. Cross-sectional areas (CSAs) of each organ in axial images of computed tomography (CT) at the L5 level in GR^fl/fl^ mice revealed CORT-induced atrophy of erector spinae muscle as well as increase in the mass of rWAT and inguinal WAT (iWAT); all of these changes were mitigated in GR-mKO mice (Figure 1, J and K). These findings clearly indicate that muscle GR signaling is a key node for changes in body composition, including muscle atrophy and increases in liver and WAT mass, in the CORT-induced obesity model.

**Figure 2:**
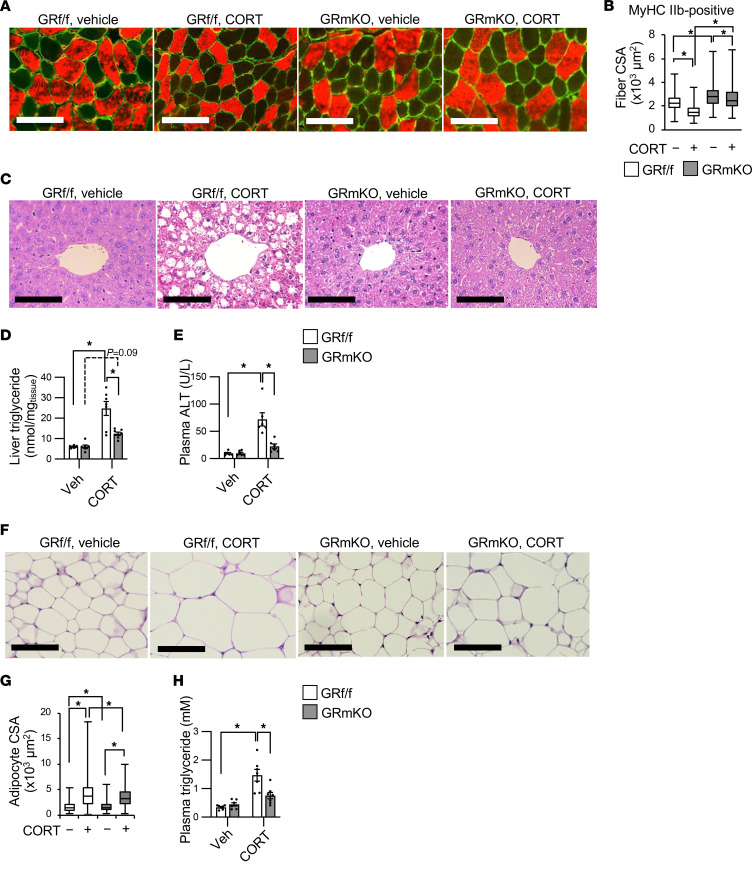
Deletion of skeletal muscle GR inhibits type IIb fiber atrophy and lipid accumulation in adipose tissue and liver in obesity induced by chronic CORT treatment. (**A**) Immunostaining of the type IIb myosin heavy chain (MyHC) (red) and laminin (green) using transverse cryosections of gastrocnemius from GR^fl/fl^ and GR-mKO male mice treated with vehicle or CORT for 4 weeks. (**B**) Cross-sectional areas (CSAs) of type IIb MyHC-positive myofibers shown as box-and-whisker plots. One hundred fibers from each animal (*n* = 3–4) were counted. (**C**) Representative images of H&E staining of liver from GR^fl/fl^ and GR-mKO mice treated with vehicle or CORT for 4 weeks. (**D**) Triglyceride concentrations in the liver. Data presented as mean ± SEM (*n* = 7). (**E**) Plasma alanine aminotransferase (ALT) levels in GR^fl/fl^ and GR-mKO mice treated with vehicle or CORT for 4 weeks. Data presented as mean ± SEM (*n* = 6). (**F**) Representative images of H&E staining of rWAT from GR^fl/fl^ and GR-mKO mice treated with vehicle or CORT for 4 weeks. (**G**) CSAs of adipocytes shown as box-and-whisker plots. One hundred cells from each animal (*n* = 4) were counted. (**H**) Plasma triglyceride levels of GR^fl/fl^ and GR-mKO mice treated with vehicle or CORT for 4 weeks. Data presented as mean ± SEM (*n* = 7). Scale bars: 100 μm (**A**, **C**, and **F**). *P < 0.05 determined by 2-way ANOVA with Tukey-Kramer post hoc test.

To clarify the changes in body composition in detail, we performed histological analyses of each organ after the 4-week CORT treatment. Analyses of average fiber CSAs in gastrocnemius muscle by each type of myosin heavy chain (i.e., MyHC I, IIa, and IIb) revealed that CORT treatment particularly induced type IIb fiber atrophy in GR^fl/fl^ mice (a 37% reduction between the medians of vehicle-treated GR^fl/fl^ and CORT-treated GR^fl/fl^ mice); such CORT-induced type IIb fiber atrophy was reduced in GR-mKO mice (a 13% reduction between the medians of vehicle-treated GR-mKO and CORT-treated GR-mKO mice) (Figure 2, A and B, and Supplemental Figure 2A). A CORT-induced shift in the peak of CSA distribution in type IIb fibers in GR^fl/fl^ mice was blunted in GR-mKO mice (Supplemental Figure 2B). Considering that muscles rich in type IIb fibers, such as gastrocnemius muscle, have higher glucocorticoid sensitivity (7), these results suggest that muscle atrophy in this obesity model mainly results from accelerated glucocorticoid signaling via GR in muscles rich in type IIb fibers. Histological analyses of liver revealed that a CORT-induced increase in lipid droplets observed in GR^fl/fl^ mice was not apparent in GR-mKO mice (Figure 2C). Consistent with this finding, the CORT-induced increase in triglyceride (TG) levels in liver was significantly reduced in GR-mKO mice (Figure 2D). A CORT-induced increase in plasma alanine aminotransferase (ALT) levels in GR^fl/fl^ mice was also reduced in GR-mKO mice (Figure 2E), suggesting that liver injury following CORT-induced lipid accumulation is mainly mediated by muscle GR signaling. Analyses of WAT revealed that the average CSA of adipocytes in rWAT increased with CORT treatment in GR^fl/fl^ mice, but this increase was ameliorated in GR-mKO mice (Figure 2, F and G). Moreover, the CORT-induced elevation of TG levels in the blood was strongly reduced in GR-mKO mice (Figure 2H). Taken together, these results show that muscle GR signaling contributes to systemic lipid storage involving the liver, WAT, and the bloodstream and to type IIb myofiber atrophy in the CORT-induced obesity mouse model.

### Muscle GR signaling is a hub connecting systemic metabolic changes in CORT-induced obesity in mice.

**Figure 3:**
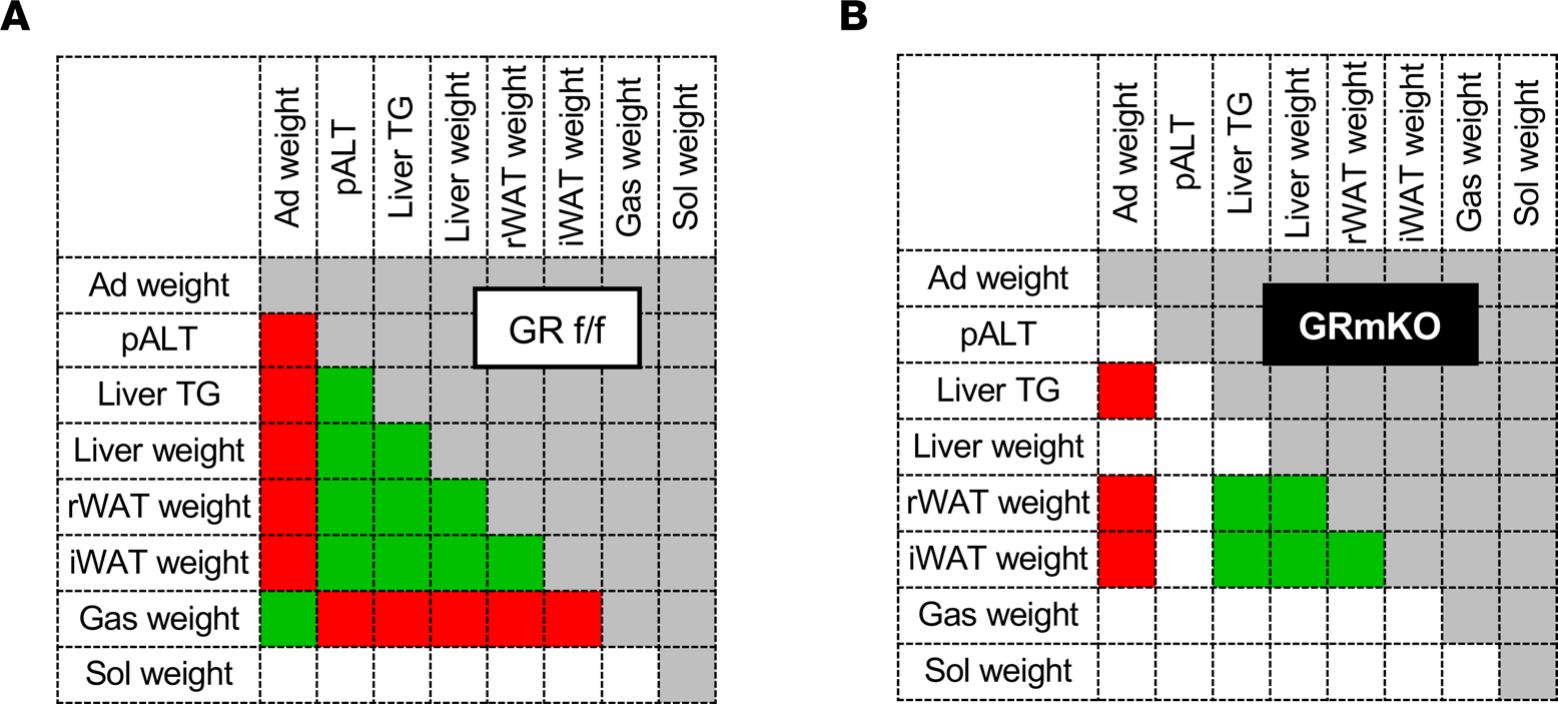
Interrelationships of systemic metabolic changes under chronic CORT treatment are coordinated by muscle GR. Correlations between metabolic parameters and tissue weights in GR^fl/fl^ (**A**) and GR-mKO mice (**B**) are shown as heatmaps (*n* = 5–6). Green or red colored cells indicate significant positive or negative correlations between each pair, respectively (*P* < 0.05). An absence of color means no correlation. Pearson’s product-moment correlation analyses were performed. Ad, adrenal gland; pALT, plasma alanine aminotransferase; TG, triglyceride; rWAT, retroperitoneal white adipose tissue; iWAT, inguinal white adipose tissue; Gas, gastrocnemius muscle; Sol, soleus muscle.

We hypothesized that the correlations among various metabolic parameters in CORT-induced obesity (22) would disappear in GR-mKO mice if these parameters depend on muscle GR signaling. These correlations in CORT- and vehicle-treated mice are presented in Figure 3A (GR^fl/fl^) and Figure 3B (GR-mKO) (each plot is indicated in Supplemental Figure 3, A and B). As expected, the weight of adrenal glands in GR^fl/fl^ mice was negatively correlated with plasma ALT levels, liver TG levels, WAT weight, and liver weight, and was positively correlated with the weight of gastrocnemius muscle, but not that of soleus muscle (Figure 3A). All parameters that were correlated with the weight of the adrenal gland were also significantly correlated with each other (Figure 3A). Taken together, the effects of CORT treatment are reflected in various systemic metabolic properties, which are in conjunction with each other. In contrast, the weight of soleus muscle was not significantly correlated with any other parameter (Figure 3A), indicating that especially GR-sensitive skeletal muscle is critical for the associations in systemic metabolism in this CORT-induced obesity model. On the other hand, the correlations among metabolic parameters in GR-mKO mice were markedly altered; the weight of the adrenal gland was not significantly correlated with plasma ALT levels, liver weight, and gastrocnemius muscle weight (Figure 3B). Moreover, most correlations between these parameters were not significant or weaker in GR-mKO mice (Figure 3B and Supplemental Figure 3B) than in GR^fl/fl^ mice (Figure 3A and Supplemental Figure 3A). Contrastingly, the weight of liver and liver TG contents both correlated with the WAT weight, even in GR-mKO mice (Figure 3B), possibly reflecting the interorgan communication occurring between WAT and liver independently of muscle GR under the activation of systemic GR signaling, as discussed later.

These results confirmed that muscle GR is a hub of interorgan communication involving muscle, liver, and WAT, leading to central obesity and metabolic dysregulation.

### Muscle GR signaling alters the muscle transcriptome and the blood amino acid, glucose, and insulin levels in CORT-induced obesity.

**Figure 4:**
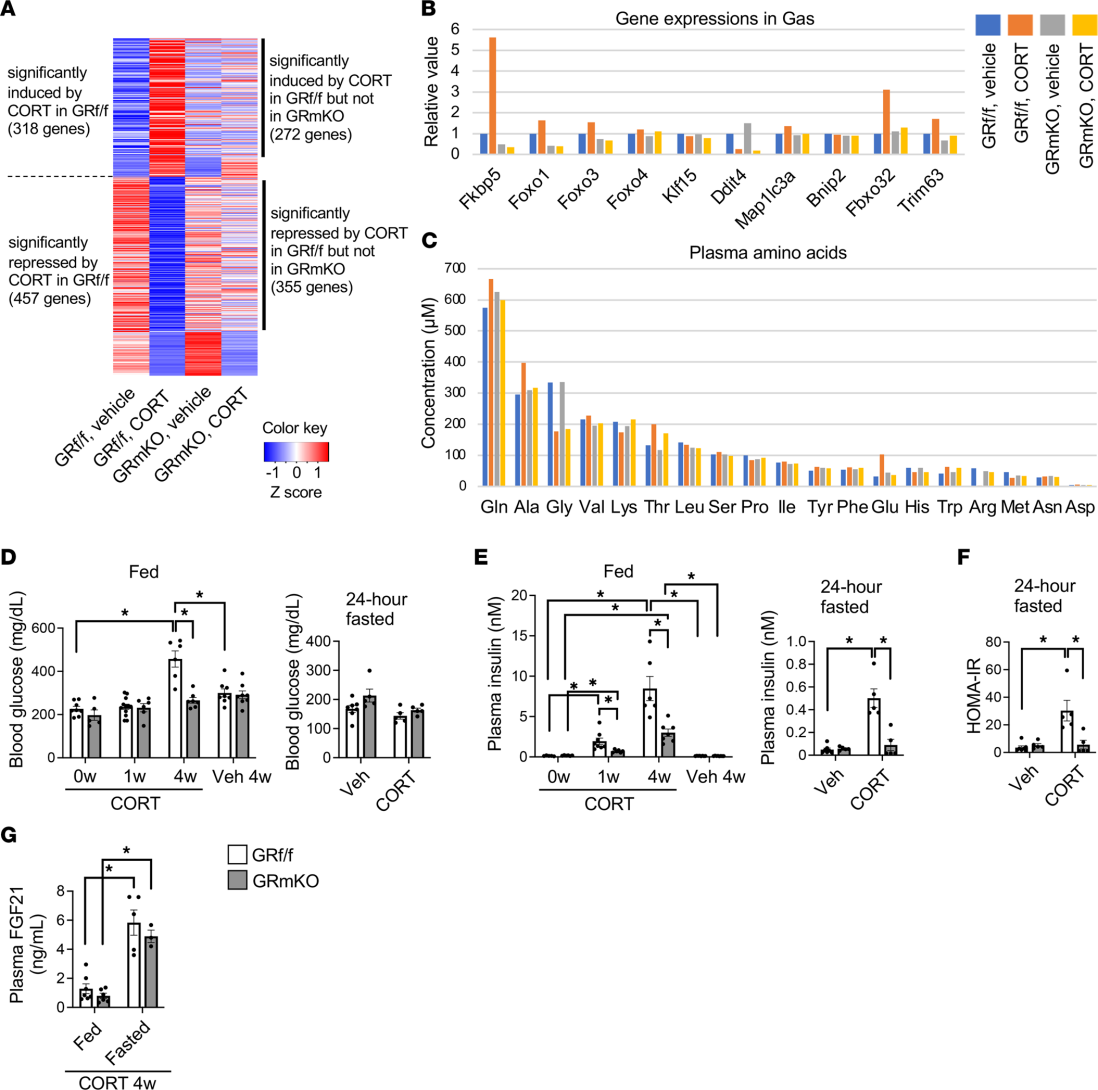
Muscle GR regulates the muscle transcriptome, alters plasma amino acid levels, and causes hyperglycemia and hyperinsulinemia in an obesity model induced by chronic CORT treatment. (**A**) RNA-seq was performed using pooled RNA samples (*n* = 3) of gastrocnemius muscle (Gas) from GR^fl/fl^ and GR-mKO mice treated with vehicle or CORT for 4 weeks. The levels of genes significantly induced or repressed by CORT in GR^fl/fl^ mice are shown as a heatmap. (**B**) The levels of some GR downstream genes are shown. (**C**) Plasma amino acid levels from GR^fl/fl^ and GR-mKO mice treated with vehicle or CORT for 4 weeks. Pooled plasma samples (*n* = 3) were used. (**D**) Blood glucose and (**E**) plasma insulin levels of fed or 24-hour-fasted GR^fl/fl^ and GR-mKO mice treated with vehicle or CORT for 0, 1, or 4 weeks. In 24-hour-fasted mice, the levels at 4 weeks were evaluated. *n* = 5–12 fed in **D**; *n* = 5–7 fasted in **D**; *n* = 5–8 fed in **E**; *n* = 5–7 fasted in **E**. (**F**) A homeostasis model assessment for insulin resistance (HOMA-IR) of 24-hour-fasted GR^fl/fl^ and GR-mKO mice treated with vehicle or CORT for 4 weeks (*n* = 5–7). (**G**) Plasma FGF21 levels in GR^fl/fl^ and GR-mKO mice treated with vehicle or CORT for 4 weeks. The results under fed or 24-hour-fasted states are shown (*n* = 3–7). Data presented as mean ± SEM. In **D**–**G**, 2-way ANOVA with Tukey-Kramer post hoc test was performed. *P < 0.05. For the fed data in **D** and **E**, statistical differences were assessed in CORT at 0 weeks (0w) versus CORT 1w, CORT 0w versus CORT 4w, and Veh 4w versus CORT 4w in each genotype, and in GR^fl/fl^ versus GR-mKO under each condition. In the fed data in **E**, data were log-transformed before statistical analysis.

We next attempted to identify the mediator(s) connecting muscle GR signaling and systemic lipid accumulation in the CORT-induced obesity model. To obtain clues, we first focused on the role of muscle GR as a transcription factor and examined CORT-induced and muscle GR–dependent transcriptomic changes in skeletal muscle. In GR^fl/fl^ mice, CORT treatment altered the expression levels of numerous genes in gastrocnemius muscle; some of these were not altered in GR-mKO mice (Figure 4A, Supplemental Figure 4, A–D, and Supplemental Tables 1–3). Notably, the levels of the well-known GR downstream genes *Fkbp5*, *Foxo3*, *Atrogin1/Fbxo32*, and *MuRF1/Trim63* (23) were upregulated by CORT in a muscle GR–dependent manner (Figure 4B). *Foxo3*, *Atrogin1*, and *MuRF1* are involved in muscle proteolysis (23); enhanced proteolysis in skeletal muscle leads to the secretion of free amino acids, especially in the form of alanine or glutamine, into the bloodstream (7, 23, 24). As expected, CORT-treated GR^fl/fl^ mice exhibited increased plasma alanine and glutamine levels, which were not observed in GR-mKO mice (Figure 4C). These changes were in clear contrast to the obesity-induced decrease in glycine (25) in both genotypes (Figure 4C), suggesting that GR-dependent muscle proteolysis supplies specific amino acids to the whole body. Alanine and glutamine may contribute to obesity-related hyperglycemia via gluconeogenesis (26). In fact, blood glucose levels in the fed state were elevated in GR^fl/fl^ mice but not in GR-mKO mice after the 4-week CORT treatment (Figure 4D). In addition, CORT-treated GR^fl/fl^ mice exhibited hyperinsulinemia, which was ameliorated in CORT-treated GR-mKO mice (Figure 4E). The CORT-induced increase in homeostasis model assessment for insulin resistance (HOMA-IR) was suppressed in GR-mKO mice after the 4-week CORT treatment (Figure 4F), indicating reduced systemic insulin resistance in CORT-treated GR-mKO mice compared with CORT-treated GR^fl/fl^ mice.

To further explore the mechanisms involved in the muscle GR–mediated systemic insulin resistance, we focused on early changes in plasma insulin levels. After 1-week CORT treatment, when CORT-induced hyperinsulinemia and its reduction in GR-mKO mice were already observed (Figure 4E), an intraperitoneal insulin tolerance test (IPITT) implied CORT-induced insulin resistance had not improved in GR-mKO mice (Supplemental Figure 5, A and B). In addition, an intraperitoneal glucose tolerance test (IPGTT) after 1-week CORT treatment showed no differences between GR^fl/fl^ and GR-mKO mice (Supplemental Figure 5C). These findings indicate that muscle GR contributes to the enhancement of insulin secretion in response to CORT before the induction of insulin resistance. Furthermore, CORT-induced systemic insulin resistance independent of muscle GR was thought to precede CORT-induced systemic insulin resistance that was dependent on muscle GR. The enhancement of hyperinsulinemia via muscle GR after 1-week CORT treatment could be derived from an increased amino acid supply from skeletal muscle.

On the other hand, an increase in plasma alanine not only accelerates gluconeogenesis in liver but also suppresses hepatokine FGF21 production (7). Plasma FGF21 levels of the CORT-treated group, however, were comparable between GR^fl/fl^ and GR-mKO mice in both the fed and fasted states (Figure 4G), indicating that other axes rather than hepatic FGF21 production are probably dominant regulators in this obesity model.

Overall, under chronic CORT treatment, muscle GR signaling mediates changes in the muscle transcriptome and the levels of humoral factors, including amino acids, glucose, and insulin, which are candidates for connecting muscle GR signaling and systemic fat accumulation. In addition, such changes in humoral factors are also influenced by muscle GR–independent mechanisms in this model.

### Hyperinsulinemia causes body composition changes associated with glucocorticoid signaling.

We then speculated that muscle GR–mediated changes in humoral factors may influence metabolism in liver and WAT in association with the local effects of CORT in hepatocytes and adipocytes, leading to lipid accumulation. To explore such metabolic crosstalk, we then evaluated transcriptome signatures of liver and WAT after the 4-week CORT treatment in this model. Many genes were affected by CORT treatment and influenced by muscle GR signaling (Supplemental Figures 6 and 7, and Supplemental Tables 4–9). Notably, in liver, the GR downstream gene *G6pc* (27) was induced similarly in GR^fl/fl^ and GR-mKO mice, but GR downstream genes *Pck1* (28) and *Tat* (29) were rather suppressed by CORT treatment, especially in GR^fl/fl^ mice (Supplemental Figure 8A). This suggests that some inhibitory factors can overcome GR signaling in the latter. *Pck1* and *Tat* are CREB and FOXO downstream genes (30, 31), and we noticed that certain genes reportedly upregulated (downregulated) by CREB and FOXOs were downregulated (upregulated) by CORT treatment (Supplemental Figure 8, A and B). Given that insulin is a primary regulator of CREB and FOXO transcriptional activity (30, 32), we speculated that hyperinsulinemia induced by CORT is a major factor contributing to systemic metabolic changes. In WAT, the levels of *Pnpla2*, which is downregulated by insulin (33), were lower in CORT-treated groups, and the levels of *Acaca* and *Fasn*, which are both upregulated by insulin (34), were higher in CORT-treated groups (Supplemental Figure 8C), suggesting again the involvement of hyperinsulinemia.

**Figure 5:**
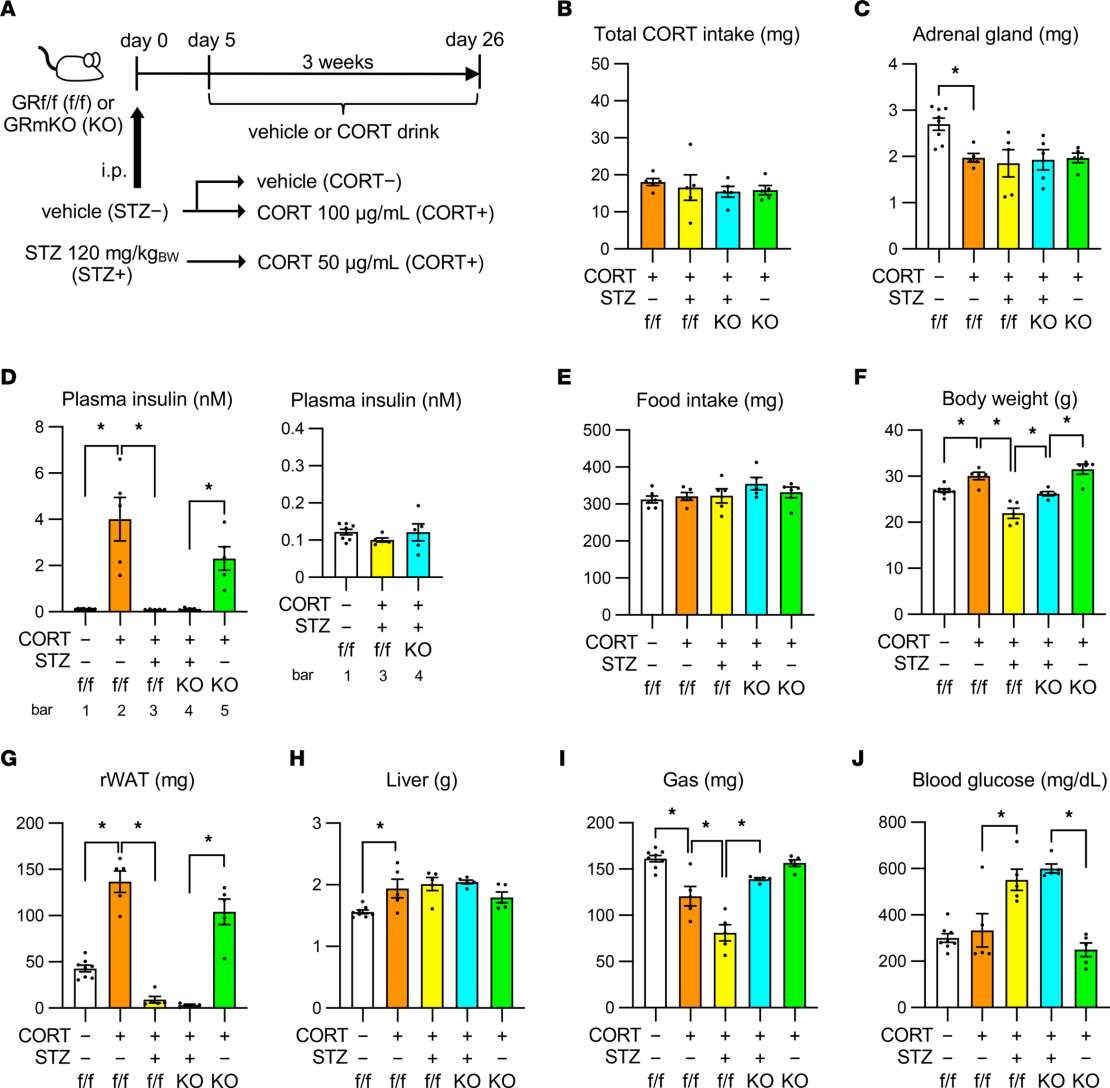
Blockade of induced hyperinsulinemia under chronic CORT treatment suppresses fat accumulation in adipose tissue, enhances muscle atrophy, and elevates blood glucose levels. (**A**) Experimental protocol. Seven-week-old male mice were pretreated with 120 mg/kg body weight (BW) STZ or vehicle (day 0) and provided drinking water with CORT or vehicle (days 5–26) ad libitum. Total CORT intake (**B**), adrenal gland weight (**C**), plasma insulin (groups with lower values are shown separately) (**D**), total food intake (**E**), BW (**F**), rWAT weight (**G**), liver (**H**), and gastrocnemius (Gas; **I**), and blood glucose levels (**J**). Data presented as mean ± SEM (*n* = 5–8). *P < 0.05 by 1-way ANOVA with Tukey-Kramer post hoc test.

To examine the role of hyperinsulinemia in metabolic changes in the CORT-induced obesity model, we administered streptozotocin (STZ) before chronic CORT treatment in GR^fl/fl^ and GR-mKO mice and evaluated their metabolic properties. Since STZ-pretreated CORT-treated (STZ+ CORT+) mice exhibited a hyperglycemia-related increase in water intake, they were provided water with half-concentration CORT solution throughout the study (Figure 5A), resulting in an equivalent intake of total CORT compared with vehicle-pretreated CORT-treated (STZ− CORT+) mice (Figure 5B). This was supported by the fact that all groups exhibited similar levels of adrenal gland atrophy (Figure 5C). Although STZ− CORT+ mice showed prominent hyperinsulinemia in this setting, STZ+ CORT+ mice did not show such an increase in plasma insulin levels, which was equivalent to those in mice pretreated with vehicle and maintained with vehicle water (STZ− CORT−) (Figure 5D). Thus, STZ+ CORT+ mice were considered to be under chronic activation of GR signaling but devoid of hyperinsulinemia-related signaling in peripheral tissues.

Each group displayed comparable total food intake (Figure 5E). STZ+ CORT+ GR^fl/fl^ mice did not show CORT-induced body weight gain (Figure 5F), and their WAT weight was remarkably decreased compared with STZ− CORT+ GR^fl/fl^ mice (Figure 5G). STZ+ CORT+ GR-mKO mice also showed a prominent decrease in WAT weight compared with STZ− CORT+ GR-mKO mice. These findings demonstrate that muscle GR–independent hyperinsulinemia is required for maintaining WAT weight, whereas muscle GR–dependent hyperinsulinemia increases the weight of WAT. On the other hand, the CORT-induced increase in liver weight in GR^fl/fl^ mice was not dampened by STZ pretreatment (Figure 5H), suggesting that physiological insulin concentrations can induce liver weight gain under CORT treatment. Moreover, the liver weight in STZ+ CORT+ GR-mKO mice tended to increase compared with STZ− CORT+ GR-mKO mice (Figure 5H), indicating that muscle GR–independent hyperinsulinemia is necessary for the inhibition of CORT-induced increase in the liver weight. Interestingly, the weight of gastrocnemius muscle further decreased in STZ+ CORT+ GR^fl/fl^ mice compared with STZ− CORT+ GR^fl/fl^ mice (Figure 5I), demonstrating that hyperinsulinemia plays a counteracting role in CORT-induced muscle atrophy. In addition, STZ+ CORT+ GR-mKO mice exhibited slightly decreased but relatively preserved muscle mass compared with STZ− CORT+ GR-mKO mice (Figure 5I), indicating that muscle GR signaling is, at least to some extent, a vital cascade when counteracting muscle atrophy by hyperinsulinemia, as discussed later. Despite maintained muscle mass, STZ+ CORT+ GR-mKO mice exhibited hyperglycemia (Figure 5J), implying that muscle GR–independent hyperinsulinemia plays an indispensable role in maintaining normal glucose levels.

Of course, the results from the inhibition of hyperinsulinemia cannot be strictly separated from the secondary effects, e.g., hyperglycemia. Nevertheless, our findings demonstrate that, under CORT-induced metabolic changes, hyperinsulinemia is a critical factor in increasing the weight of WAT, probably via adipogenesis and/or lipogenesis. In more detail, mild hyperinsulinemia is necessary, even under muscle GR deficiency, for compensatory increases in the weight of WAT, and may therefore inhibit increases in liver weight and maintain normoglycemia. On the other hand, muscle GR signaling enhances hyperinsulinemia, promoting increases in the weight of WAT.

### Insulin suppresses muscle glucocorticoid signaling, leading to changes in atrogene expression.

One of the important findings in Figure 5 is that STZ-induced reduction in the weight of gastrocnemius muscle in CORT-treated GR-mKO mice was 11%, which was lower than the 33% reduction in CORT-treated GR^fl/fl^ mice (Figure 5I). This finding was surprising because CORT-treated GR^fl/fl^ mice are starting at a much lower baseline than CORT-treated GR-mKO mice. The difference in STZ-induced muscle atrophy might be due to differences in hyperinsulinemia levels, but we speculated that hyperinsulinemia may counteract muscle atrophy by interfering with muscle GR signaling.

**Figure 6:**
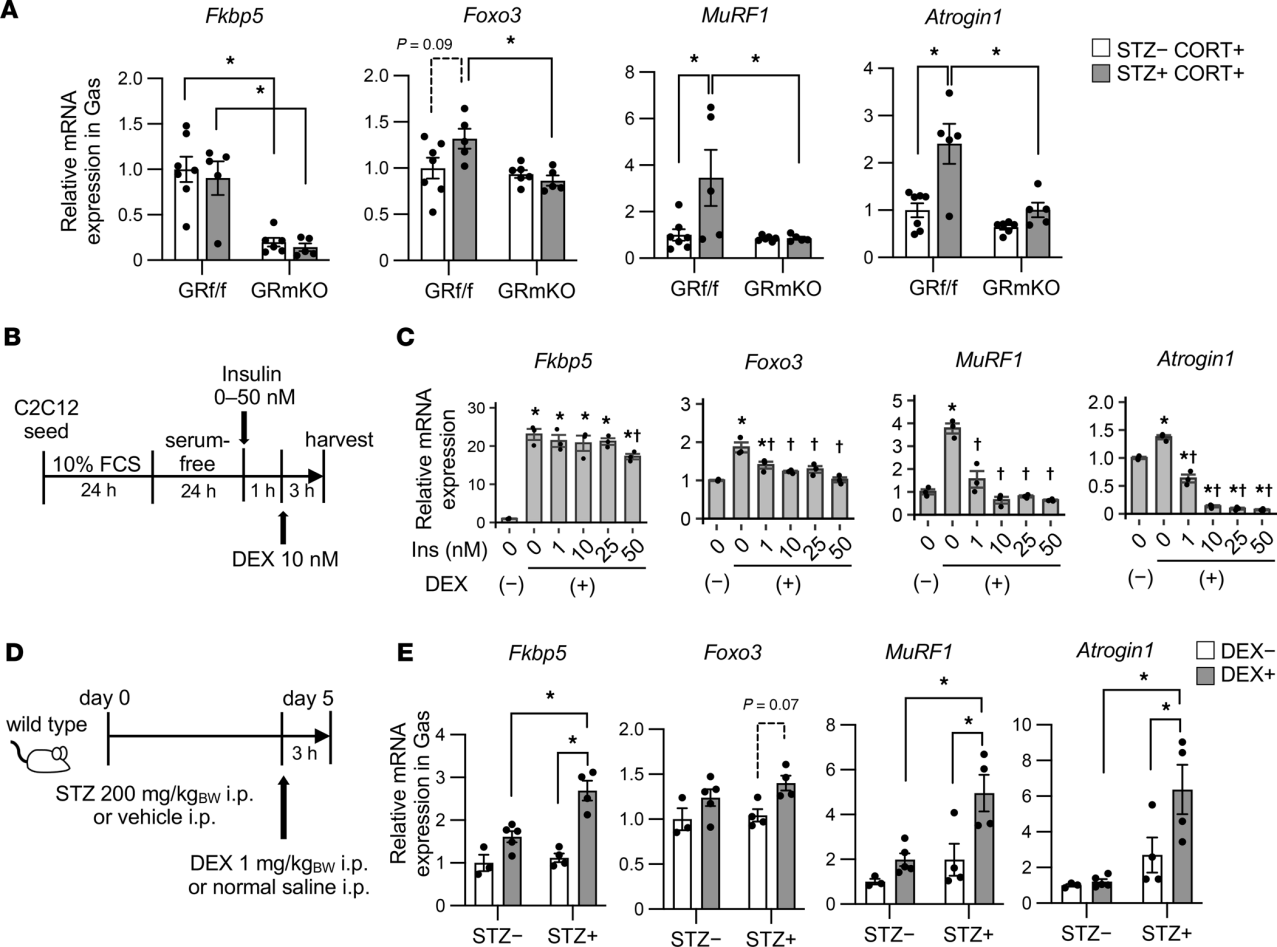
Hyperinsulinemia induced by chronic CORT treatment counteracts muscle atrophy by inhibiting muscle GR downstream gene expression. (**A**) The gene expression of gastrocnemius muscle (Gas) from GR^fl/fl^ or GR-mKO male mice pretreated with streptozotocin (STZ) or vehicle (day 0) and provided drinking water with CORT (day 5–26) ad libitum, as indicated in Figure 5A. Data presented as mean ± SEM (*n* = 5–7). *P < 0.05 by 2-way ANOVA with Tukey-Kramer post hoc test. (**B** and **C**) C2C12 myoblasts were pretreated with insulin at the indicated concentrations and followed by stimulation with 10 nM dexamethasone (DEX). The experimental protocol (**B**) and gene expression levels (**C**). Data presented as mean ± SEM (*n* = 3). *P < 0.05 versus DEX (−) after 0 nM insulin treatment; †*P* < 0.05 versus DEX (+) after 0 nM insulin treatment. Significance determined by 1-way ANOVA and Dunnett’s post hoc test. (**D** and **E**) Seven-week-old male mice were pretreated with 200 mg/kg BW STZ or vehicle (day 0) and followed by injection of 1 mg/kg BW DEX or normal saline (day 5). The experimental protocol (**D**). mRNA levels for Gas in each group (**E**). Data presented as mean ± SEM (*n* = 3−5). *P < 0.05 by 2-way ANOVA with Tukey-Kramer post hoc test.

To verify the insulin effect on muscle GR signaling, we examined the target genes regulated by GR (*Fkbp5*, *Foxo3*, *MuRF1*, and *Atrogin1*) in gastrocnemius muscle. The levels of *Foxo3*, *MuRF1*, and *Atrogin1* in STZ+ CORT+ GR^fl/fl^ mice were higher or tended to be higher than those in STZ− CORT+ GR^fl/fl^ mice, while the expression level of *Fkbp5* was similar between them (Figure 6A). The levels of *Foxo3*, *MuRF1*, and *Atrogin1* in gastrocnemius muscle from GR-mKO mice, however, showed no increase or only a mild increase with STZ pretreatment (Figure 6A). These results support the findings of STZ-induced alterations in muscle mass (Figure 5I) and indicate that hyperinsulinemia inhibits GR-dependent increases in atrogene expression in skeletal muscle.

To examine whether such counteracting effects on muscle GR signaling result from the direct action of insulin on myocytes, we evaluated the insulin action on dexamethasone-induced (DEX-induced) expression of GR-regulated genes in C2C12 cells (Figure 6B). We found that increased *Fkbp5* expression was not suppressed, although slight suppression was caused by pretreatment with 50 nM insulin. In contrast, the increased expression of *Foxo3*, *MuRF1*, and *Atrogin1* was dose-dependently inhibited by pretreatment with insulin (Figure 6C). The levels of *Atrogin1* when pretreated at 1 nM or higher concentrations of insulin were lower than those with no insulin pretreatment (Figure 6C). These results indicate that the expression of GR downstream genes in myocytes is suppressed by insulin, and that insulin sensitivity differs among genes.

To further validate the suppressive effects of insulin against muscle GR signaling, we also examined acutely induced gene expression with a single shot of intraperitoneal DEX after STZ pretreatment (Figure 6D). Compared with vehicle-pretreated mice, STZ-pretreated mice exhibited lower plasma insulin and higher blood glucose levels, both of which were not largely disturbed by DEX injection (Supplemental Figure 9, A and B). Injection of DEX tended to promote the expression of *Fkbp5*, *Foxo3*, *MuRF1*, and *Atrogin1* in gastrocnemius muscle, but this induction was more evident in STZ-pretreated mice (Figure 6E), which again demonstrated the counteracting effects of insulin on GR signaling in skeletal muscle. The weight of gastrocnemius muscle was lower or tended to be lower in STZ-pretreated mice than in vehicle-pretreated mice, although the weight of soleus muscle did not significantly change (Supplemental Figure 9C). These results support the notion that glucocorticoid-sensitive muscle is susceptible to alterations in insulin signaling.

Collectively, we concluded that hyperinsulinemia induced by chronic CORT treatment suppresses further muscle atrophy through interfering with muscle GR signaling and regulating atrogene levels. This effect of hyperinsulinemia may not be entirely attributable to the direct action of insulin on skeletal muscle, since hyperglycemia-induced muscle atrophy, for example, may also be involved (35). Nevertheless, our findings present evidence that muscle GR signaling can be a critical node of insulin-mediated counteraction against muscle atrophy.

### Muscle GR signaling accelerates glucose intolerance and obesity in ob/ob mice.

**Figure 7:**
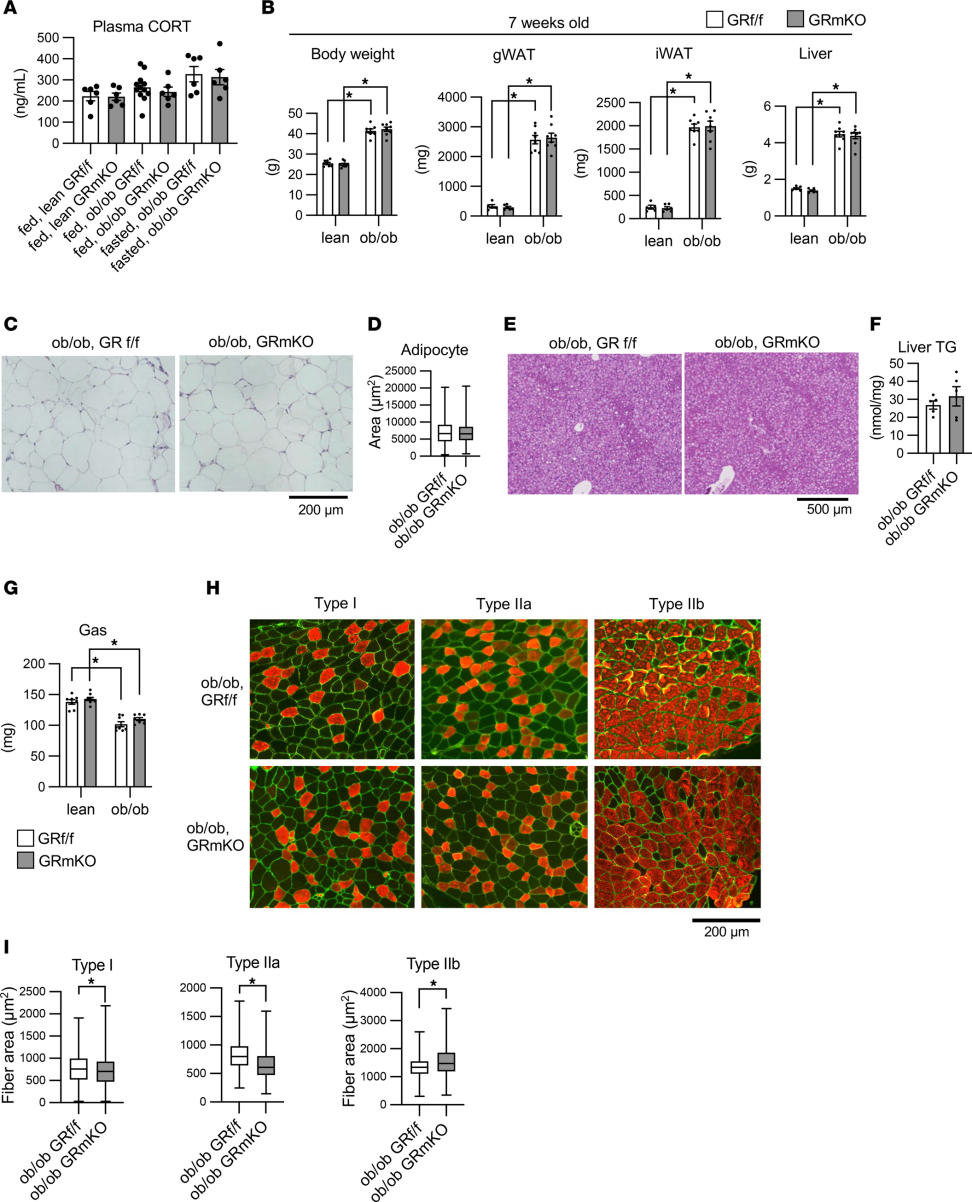
Muscle-specific GR knockout does not suppress fat accumulation in WAT or liver but leads to histological changes in skeletal muscle in 7-week-old *ob/ob* mice. (**A**) Plasma CORT levels of 7-week-old male mice under a fed or 16-hour fasted state (*n* = 6−12). (**B**) Tissue weight of 7-week-old male mice (*n* = 5−8). (**C**) H&E staining of retroperitoneal WAT of 7-week-old male mice and (**D**) the distribution of the adipocyte sizes. CSAs of adipocytes are shown as box-and-whisker plots. One hundred cells from each animal (*n* = 4) were counted. (**E**) H&E staining of liver of 7-week-old male mice. (**F**) Triglyceride concentrations in the liver (*n* = 5). (**G**) Gastrocnemius (Gas) weight of 7-week-old male mice (*n* = 8−9). (**H**) Immunostaining for MyHCs in Gas from 7-week-old male mice. MyHC type I, IIa, or IIb (red) and laminin (green). Regions enriched in each fiber type are shown as representative images. (**I**) Fiber type–specific CSA. All fibers positive for each MyHC were quantified using 3 mice from each group. If more than 200 fibers were present, 200 fibers were quantified. Scale bars: 200 μm (**C** and **H**) and 500 μm (**E**). Data presented as mean ± SEM. *P < 0.05 by 2-way ANOVA with Tukey-Kramer post hoc test (**A**, **B**, and **G**), Mann-Whitney *U* test (**D** and **I**), or 2-tailed Welch’s *t* test (**F**).

Finally, we evaluated whether muscle GR signaling under physiological levels of plasma CORT also contributes to hyperinsulinemia and obesity. We generated GR-mKO mice in the *ob/ob* background (*ob/ob* GR-mKO mice) and compared them to their control (*ob/ob* GR^fl/fl^) mice (Supplemental Figure 10A). We confirmed that the leptin-deficient phenotype was not lost in *ob/ob* GR-mKO mice (Supplemental Figure 10B), and that a muscle GR–deficient phenotype was observed in *ob/ob* GR-mKO mice (Supplemental Figure 10, C and D). An increase in food intake was equally observed in both *ob/ob* GR^fl/fl^ and *ob/ob* GR-mKO mice (Supplemental Figure 11A). Plasma CORT levels were similar in lean and *ob/ob* mice and were not significantly different between *ob/ob* GR^fl/fl^ and *ob/ob* GR-mKO mice (Figure 7A). At 7 weeks of age, the body weight, WAT weight, and liver weight were higher in *ob/ob* mice, with no differences between *ob/ob* GR^fl/fl^ and *ob/ob* GR-mKO mice (Figure 7B). Histological analyses implied no significant differences between *ob/ob* GR^fl/fl^ and *ob/ob* GR-mKO mice with respect to fat accumulation in WAT (Figure 7, C and D). Similarly, liver TG content did not differ between *ob/ob* GR^fl/fl^ and *ob/ob* GR-mKO mice (Figure 7, E and F). The gene expression related to chronic inflammation in WAT was induced equally in *ob/ob* GR^fl/fl^ and *ob/ob* GR-mKO mice (Supplemental Figure 11B), supporting the minor contribution of muscle GR to metabolism in WAT at this age. The weight of gastrocnemius muscle, which was lower in *ob/ob* mice than in lean mice, was not statistically different between *ob/ob* GR^fl/fl^ and *ob/ob* GR-mKO mice (Figure 7G). However, *ob/ob* GR-mKO mice showed slight histological changes, including increased CSAs of type IIb fibers and decreased CSAs of type I and IIa fibers, compared with *ob/ob* GR^fl/fl^ mice (Figure 7, H and I). These changes suggest that muscle GR signaling under leptin deficiency can affect local metabolism in muscle.

**Figure 8:**
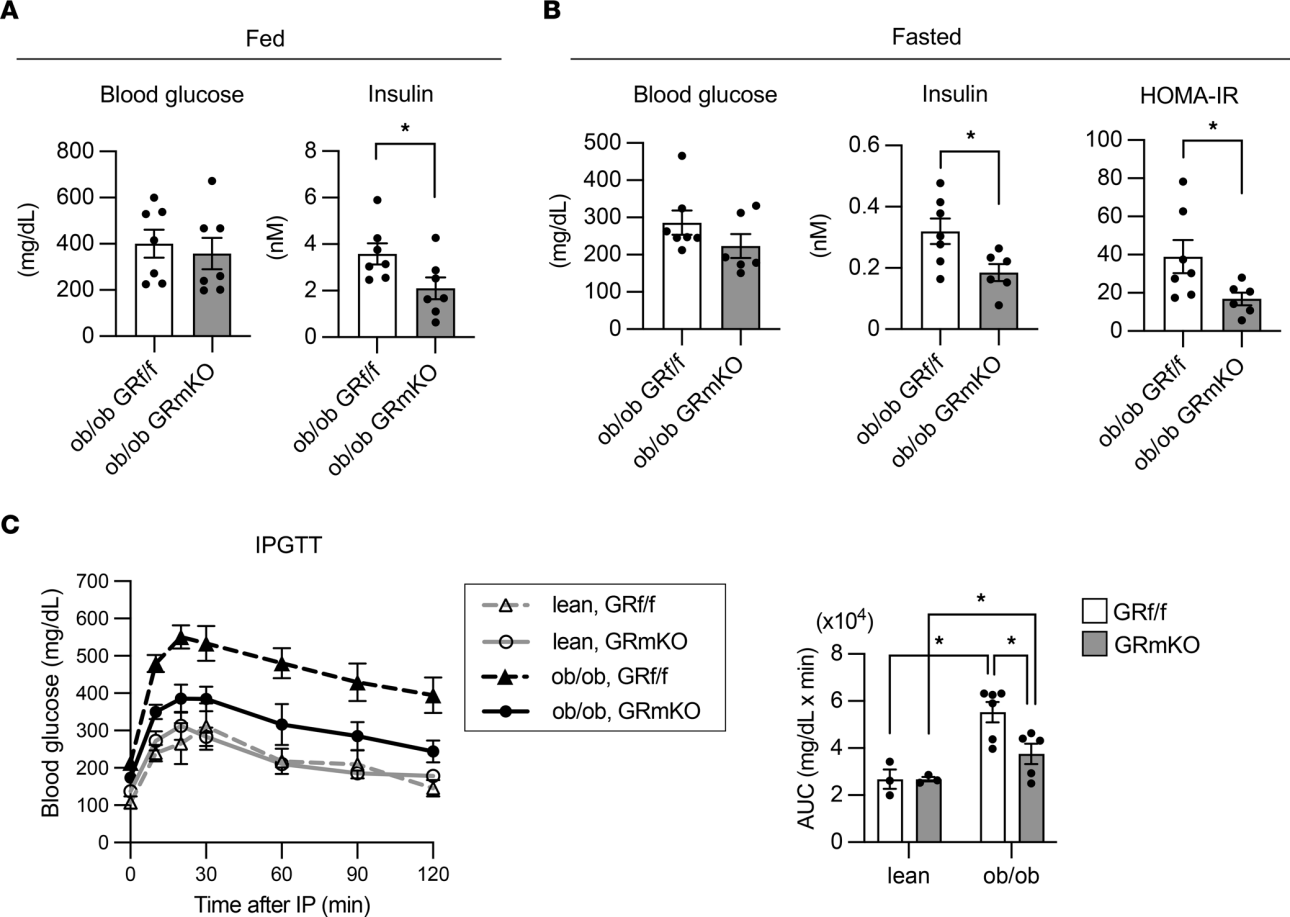
Muscle-specific GR knockout reduces hyperinsulinemia and glucose intolerance in 7-week-old *ob/ob* mice. (**A**) The blood glucose and plasma insulin levels under the fed state (*n* = 7). (**B**) The blood glucose and plasma insulin levels, and HOMA-IR under 16-hour fasted state (*n* = 6−7). (**C**) The results of the intraperitoneal glucose tolerance test (IPGTT). Seven-week-old mice after 16 hours of fasting were intraperitoneally injected with 1 g/kg BW glucose. Blood glucose levels at 0, 10, 20, 30, 60, 90, and 120 minutes: lean GR^fl/fl^ (*n* = 3), lean GR-mKO (*n* = 3), *ob/ob* GR^fl/fl^ (*n* = 6), and *ob/ob* GR-mKO (*n* = 5). The area under the curve (AUC) is also shown. Data presented as mean ± SEM. *P < 0.05 by 2-tailed Welch’s *t* test (**A** and **B**; insulin, HOMA-IR), Mann-Whitney *U* test (**B**, blood glucose), or 3-way repeated-measures ANOVA (**C**, blood glucose): *P* < 0.001 (time), *P* < 0.001 (obesity), *P* = 0.073 (genotype), *P* = 0.071 (time × obesity), *P* = 0.296 (time × genotype), *P* = 0.036 (obesity × genotype), *P* = 0.588 (time × obesity × genotype). Two-way ANOVA with Tukey-Kramer post hoc test was used in **C** (AUC).

Given the muscle GR–mediated enhancement of hyperinsulinemia in the CORT-induced obesity model, we next examined the plasma insulin levels of the *ob/ob* model. Strikingly, plasma insulin levels were lower in *ob/ob* GR-mKO mice than *ob/ob* GR^fl/fl^ mice, and *ob/ob* GR-mKO mice showed reduced HOMA-IR (Figure 8, A and B). The blood glucose levels in IPGTT also demonstrated improved glucose tolerance in *ob/ob* GR-mKO mice (Figure 8C). Muscle atrogenes such as *MuRF1/Trim63* and *Atrogin1/Fbxo32* were downregulated in *ob/ob* GR^fl/fl^ mice compared with lean GR^fl/fl^ mice (Supplemental Figure 11C), possibly reflecting the counteracting role of hyperinsulinemia in *ob/ob* mice for maintaining muscle mass. In addition, some gene expression changes in gastrocnemius muscle induced by obesity were not observed in GR-mKO mice (Supplemental Figure 11, D–F, and Supplemental Tables 10–12). Therefore, the inhibition of muscle GR signaling under progression of obesity mitigates hyperinsulinemia without apparent changes in fat accumulation in WAT or in liver, possibly via changes in local muscle metabolism.

**Figure 9:**
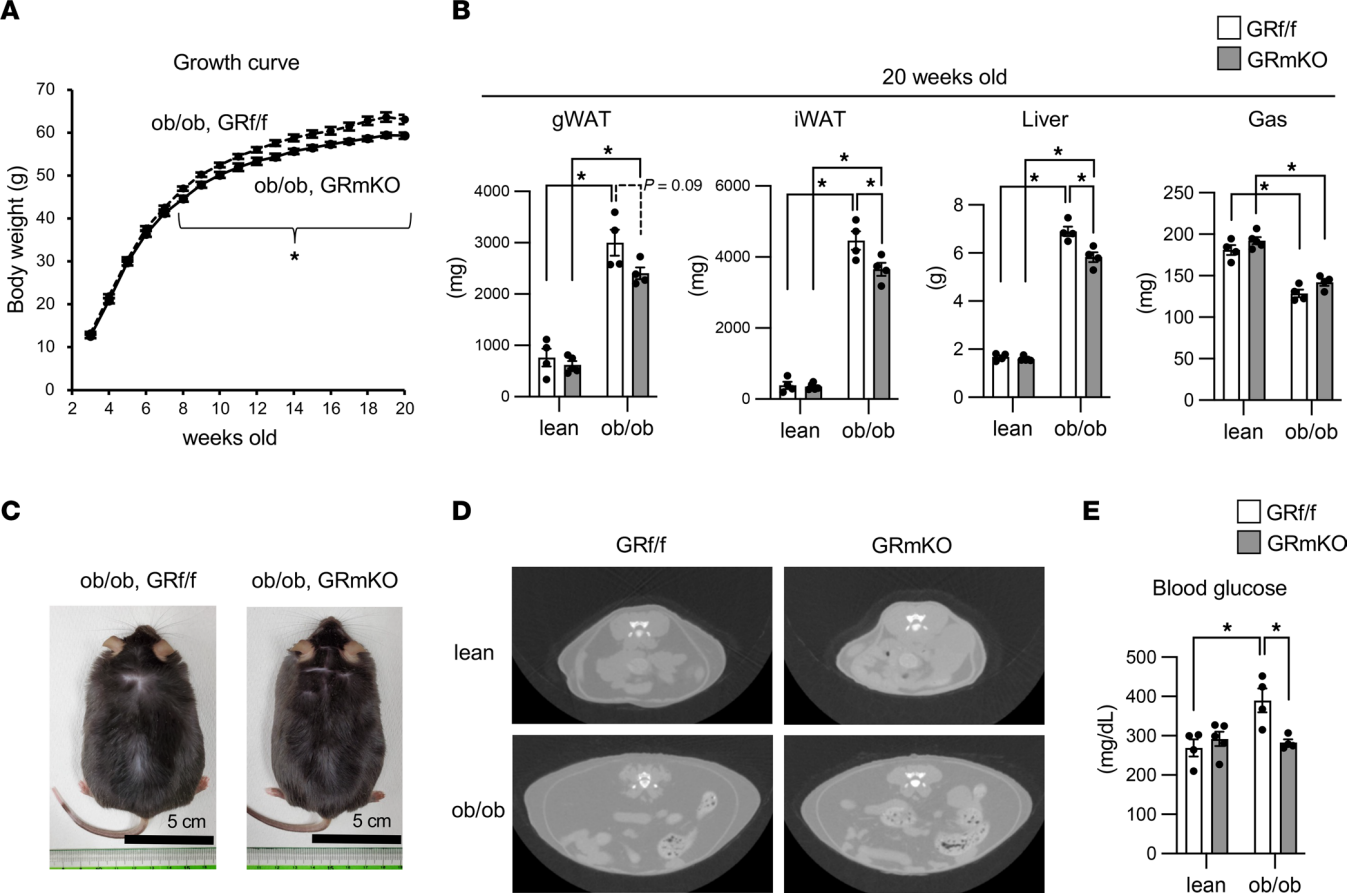
Muscle-specific GR knockout inhibits obesity progression and hyperglycemia in *ob/ob* mice. (**A**) Growth curves of *ob/ob* GR^fl/fl^ and ob/ob GR-mKO male mice. Samples used were greater than or equal to 8 mice for each group. (**B**) Tissue weight of 20-week-old mice (*n* = 4−5). (**C**) Appearance of 20-week-old *ob/ob* GR^fl/fl^ and *ob/ob* GR-mKO male mice. (**D**) Axial CT images of L5 in 20-week-old mice. (**E**) The concentration of blood glucose under ad libitum–fed conditions (*n* = 4−5). Data presented as mean ± SEM. *P < 0.05 by 2-tailed Welch’s *t* test (each time point in **A**) or 2-way ANOVA with Tukey-Kramer post hoc test (**B** and **E**).

We assumed that the metabolic changes in *ob/ob* GR-mKO mice, including suppression of hyperinsulinemia, require a long time to exert effects on the changes in systemic body composition. We therefore observed the progress of body weight and fat accumulation after 7 weeks of age in the *ob/ob* model. As expected, the increase in body weight was suppressed in *ob/ob* GR-mKO mice compared with *ob/ob* GR^fl/fl^ mice (Figure 9A). At 20 weeks of age, the weight of WAT and liver was significantly suppressed or tended to be suppressed in *ob/ob* GR-mKO mice compared with *ob/ob* GR^fl/fl^ mice, although there was a minor and nonsignificant recovery in the weight of gastrocnemius muscle (Figure 9B). The appearance of mice and axial CT images at 20 weeks old supported the reduction in fat accumulation in *ob/ob* GR-mKO mice (Figure 9, C and D). Blood glucose levels were lower in *ob/ob* GR-mKO mice than in *ob/ob* GR^fl/fl^ mice (Figure 9E), demonstrating that the inhibition of muscle GR signaling improves obesity-associated hyperglycemia.

**Figure 10:**
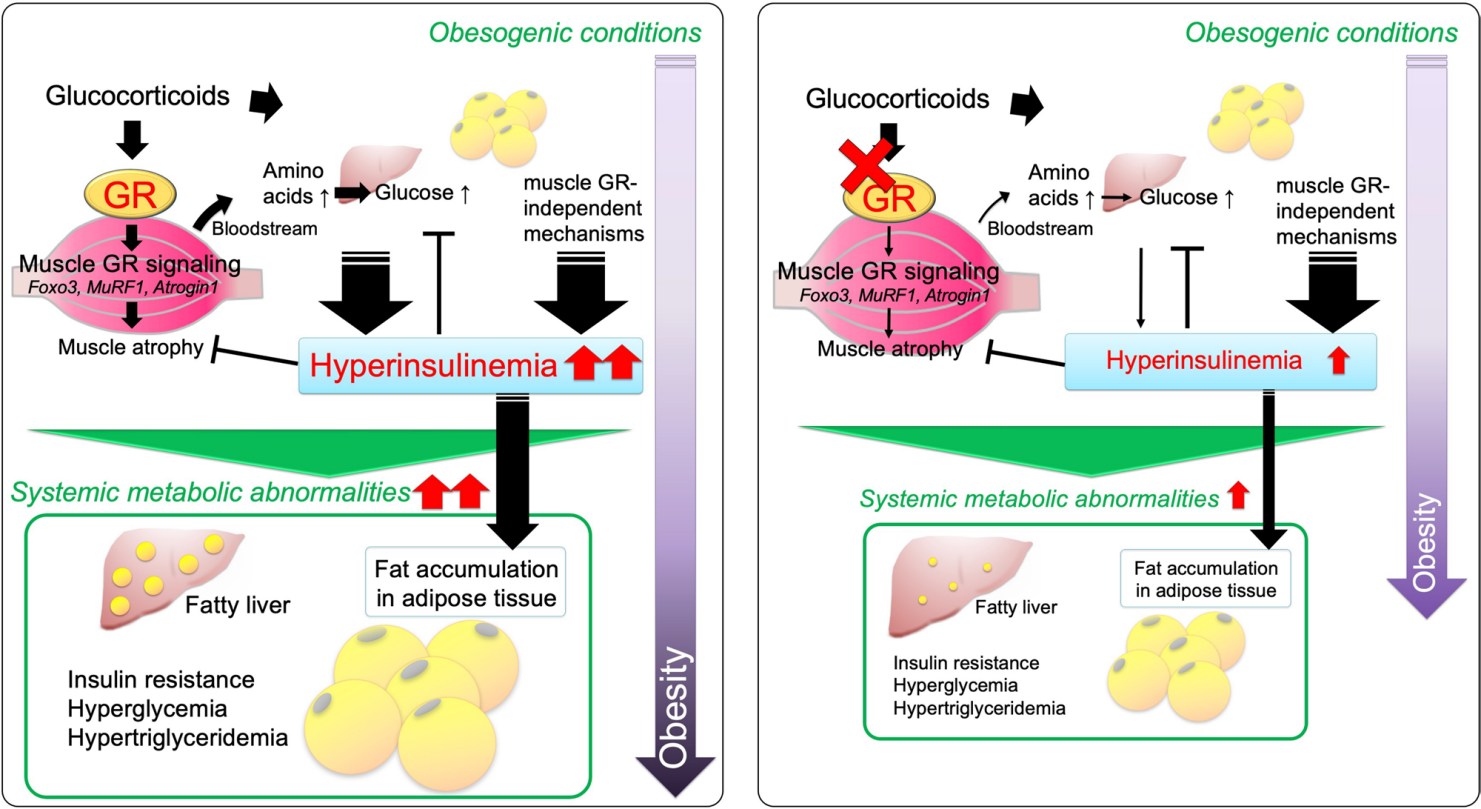
Schematic diagram of metabolic changes mediated by muscle GR signaling in obesity. Under obesogenic conditions, glucocorticoid signaling in skeletal muscle mediates systemic metabolic changes, including fat accumulation in adipose tissue, induction of fatty liver, insulin resistance, hyperglycemia, and hypertriglyceridemia (left). Deletion of muscle GR mitigates these abnormalities (right). Primarily, muscle GR signaling mediates protein catabolism, releases amino acids (substrates for glucose production), and enhances hyperinsulinemia. Hyperinsulinemia is also induced via muscle GR–independent mechanisms. Excessive hyperinsulinemia plays a role in promoting systemic metabolic abnormalities, especially for fat accumulation in adipose tissue. Hyperinsulinemia may cause obesity-related metabolic abnormalities, but a certain degree of compensatory hyperinsulinemia is necessary for the maintenance of healthy phenotypes, e.g., maintaining normoglycemia. In addition, hyperinsulinemia counteracts muscle atrophy by intervening in muscle GR signaling. Although favorable plasma insulin levels depend on individual conditions, the blockade of muscle GR signaling appropriately reduces hyperinsulinemia, inhibits subsequent systemic fat accumulation, and protects against glucose intolerance and muscle atrophy.

In summary, 2 obese mouse models under excessive or physiological CORT levels revealed that there is muscle GR–originated and insulin-mediated metabolic communication involving WAT, liver, and skeletal muscle, which is crucial to the induction of body composition changes and glucose intolerance. A schematic diagram obtained from this study is shown in Figure 10.

## Discussion

This study demonstrated that muscle GR signaling contributes to metabolic abnormalities in glucocorticoid excess, including muscle atrophy, systemic lipid accumulation, glucose intolerance, and hyperinsulinemia. We also revealed that hyperinsulinemia induced by muscle GR signaling facilitates lipid accumulation, especially in WAT, and also inhibits muscle atrophy. Another critical point demonstrated by our experiments on *ob/ob* mice is the possible involvement of muscle GR in general obesity. Despite a lack of strong positive correlations between BMI and systemic glucocorticoid levels in general human obesity (36, 37), our findings indicate that local muscle GR signaling must be more closely related to obesity and metabolic diseases than had been expected, regardless of hypercortisolemia. According to a recent systematic analysis, the transcriptomic data of skeletal muscle from patients with type 2 diabetes showed enrichment of genes related to glucocorticoid response (38). The interorgan communication, including muscle GR signaling, an evolutionarily acquired mechanism that is crucial in starvation response, can be detrimental to some context in the era of excessive eating.

The importance of local GR signaling in tissues or cells under obesogenic states has been of great interest (39). Its relevance is supported by research findings showing that mutations or polymorphisms in the GR-encoding *NR3C1* gene lead to obesity and metabolic diseases if they express a more active form of the GR protein (40–43). Along this line, the significance of tissue-specific GR signaling in obesity has been reported; for example, liver GR signaling accelerates fat accumulation in adipose tissue and worsens systemic insulin sensitivity in a 2-week DEX treatment model (15). This liver-fat interrelation is also consistent with our finding that there are residual correlations between WAT weight and the parameters associated with hepatic steatosis in GR-mKO mice after CORT treatment. Meanwhile, the significance of GR signaling in adipocytes has been noted in many studies. For example, deletion of adipocyte GR promotes a glucocorticoid-induced increase in WAT (15, 17, 44), reduces hyperinsulinemia and insulin resistance (17, 44, 45), and prevents CORT-induced hepatic steatosis (44). Our findings revealed a muscle GR–mediated increase in WAT and liver weights, which we believe is a novel mechanism regulating body composition under glucocorticoid excess. Taken together, all of the glucocorticoid signaling in skeletal muscle, WAT, and liver contribute to systemic fat accumulation via regulation of other organs. In addition, such metabolic changes based on interorgan communication are closely related to hyperinsulinemia, which could be a cause as well as a result of obesity.

Our RNA-seq analyses indicated that various genes in gastrocnemius muscle, WAT, and liver are upregulated or downregulated by CORT treatment and are influenced by muscle GR signaling. Such comprehensive changes are thought to determine systemic metabolic properties, including hyperinsulinemia. Of these, enhanced muscle protein catabolism and the efflux of amino acids, such as alanine, may induce hyperglycemia and hyperinsulinemia through promotion of amino acid metabolism in liver (26), or by the direct influence of amino acids on islets (46, 47). Such hyperinsulinemia via muscle GR may contribute to the induction of insulin resistance under chronic CORT treatment. Supporting this, our analyses showed that muscle GR can enhance insulin secretion in response to CORT before the induction of insulin resistance (Figure 4E and Supplemental Figure 5). This suggests that CORT treatment induces muscle GR–independent systemic insulin resistance initially but induces muscle GR–dependent insulin resistance via the induction of hyperinsulinemia during long-term CORT treatment. Increased amino acid supply from skeletal muscle through muscle GR is thought to play an important role in enhancing hyperinsulinemia, but other mechanisms, including impairment of muscle insulin signaling via muscle GR, may also be involved in CORT-induced obesity. In fact, it has been reported that CORT-induced impairment of insulin signaling in myocytes may be crucial (48). Such insulin resistance in local tissues may result in hyperinsulinemia, which can play a causal role in obesity (49). Notably, the blockade of muscle GR signaling in *ob/ob* mice under physiological plasma CORT levels also reduced hyperinsulinemia at 7 weeks of age, whereas they exhibited only minor recovery in muscle weight and no reduction in fat accumulation in WAT and liver, suggesting that the underlying mechanisms are not limited to substrate redistribution. Further analysis in future studies is necessary to elucidate the detailed mechanisms. In any case, although multiple factors are likely to be involved, our results provide in vivo evidence that muscle GR signaling can cause excessive hyperinsulinemia and can thereby result in obesity.

We also revealed the substantial contribution of hyperinsulinemia to counteracting glucocorticoid-induced muscle atrophy, which seems to be a negative feedback. Interestingly, muscle-specific *MuRF1*-transgenic mice show an equivalent volume of skeletal muscle along with striking hyperinsulinemia (50), which supports the intrinsic compensatory mechanism against enhanced muscle catabolic signaling. Taken together, these results show that hyperinsulinemia is a vital mechanism in preventing muscle atrophy both in the physiological state and in glucocorticoid excess. In future studies, the time-dependent manner of this compensation should be investigated to understand the pathophysiological mechanisms underlying the metabolic dysregulation involving muscle atrophy and alterations in insulin secretion capacity.

There are some limitations to this study. First, glucocorticoids are important regulators of circadian rhythm (51), but we did not assess this effect in this study. Chronic CORT treatment via drinking water may affect both the central and peripheral circadian clocks, including oscillations of gene expression; this should be addressed in future studies. Second, the significance of muscle GR signaling and related interorgan communication might be different among catabolic models (18, 52, 53), nutritional states (54, 55), species (56), and sexes (57–61). Comprehensive analyses that include an assessment of energy balance in various models at different time points should be performed to reveal conditionally determined metabolic pathways. Nevertheless, the findings in this study strengthen the importance of muscle metabolism regulated by GR signaling to combat metabolic diseases. Third, GR expression in GR-mKO mice is suppressed beginning at the development stage. Thus, compensatory mechanisms may influence their phenotypes. Future studies using inducible KO models to assess different stages of obesity can clarify the pathological roles of muscle GR signaling in greater detail. Finally, glucocorticoid effects on muscle may include those mediated by mineralocorticoid receptors (MRs) in skeletal muscle (62). Our RNA-seq data on gastrocnemius muscle showed that the transcript levels of GR are about 10 times higher than those of MR (Supplemental Tables 1 and 10), possibly indicating that MR plays a minor role. Although our study clearly demonstrates the metabolic role of muscle GR, the pathophysiological significance of the relative abundance of GR and MR should be assessed in future studies.

In conclusion, the inhibition of muscle GR signaling prevents obesity and related metabolic abnormalities, partly based on the significant suppression of hyperinsulinemia and subsequent reduction of systemic fat accumulation. The signaling cascade regulated by muscle GR is a fascinating target to combat obesity or obesity-related diseases, because not only the suppression of hyperinsulinemia and hyperglycemia but also keeping muscle mass are anticipated. The roles of muscle GR must be a clue to unravel the pathophysiology of obesity and to realize effective interventions against metabolic diseases.

## Methods

### Animal studies.

Mice were maintained in individual cages (1 mouse per cage) on a 12-hour light/dark cycle with free access to water and a stock pellet diet (CA-1, CLEA Japan, 3.47 kcal/g) in a specific pathogen–free environment at the Laboratory Animal Research Center, Institute of Medical Science, University of Tokyo, and in the Department of Biochemistry, Keio University School of Medicine. All experiments were conducted using male mice.

GR^fl/fl^ mice were provided by Günther Schütz (German Cancer Research Center, Heidelberg, Germany) (63). GR-mKO mice were generated as previously described (7). Briefly, exon 3 of the *Nr3c1* gene, which corresponds to the region containing the DNA-binding domain of GR, was floxed, and functional GR protein was deleted in a myofiber-specific manner using *Acta1*-Cre (Supplemental Figure 12). These mice were regularly backcrossed with C57BL/6J (CLEA Japan). To create a mouse model in which systemic GR signaling is chronically activated, the drinking water of 8-week-old mice was replaced with a 1% ethanol solution containing 100 μg/mL CORT (FUJIFILM Wako Pure Chemicals Corporation) or a vehicle (1% ethanol solution), and the mice were allowed to drink normally. For the experiments using STZ and CORT, 7-week-old mice were intraperitoneally administered 120 mg/kg body weight STZ (FUJIFILM Wako Pure Chemicals Corporation) dissolved in 100 mM sodium citrate buffer (pH 4.5) or sodium citrate buffer alone (vehicle) (day 0). Five days after the injection of STZ or vehicle, mice were provided water with 50 μg/mL CORT for STZ-pretreated mice or with 100 μg/mL CORT in vehicle-pretreated mice. On day 26, the mice were euthanized. Total CORT intake in each mouse was calculated by determining the decrease in the volume of drinking water. For the study using STZ and DEX, 7-week-old mice were intraperitoneally injected with 200 mg/kg body weight STZ or vehicle on day 0. Five days after the administration of STZ or vehicle, mice were intraperitoneally injected with DEX (Sigma-Aldrich) at 1 mg/kg body weight or saline and were euthanized 3 hours later. Lep^ob/+^ mice were obtained from Charles River Laboratories. To generate *ob/ob* GR^fl/fl^, *ob/ob* GR-mKO, and their control lean mice, we first crossed Lep^ob/+^ mice with GR Flox^+/+^ Cre^–/–^ (GR^fl/fl^) or GR Flox^+/+^ Cre^+/–^ (GR-mKO), all of which were offspring of mice that had been backcrossed with C57BL/6J for more than 10 generations. By crossing them and their offspring, we obtained GR Flox^+/+^ Cre^–/–^ Lep^ob/+^ and GR Flox^+/+^ Cre^+/–^ Lep^ob/+^. They were regularly crossed with GR-mKO or GR^fl/fl^ mice. Then, we crossed GR Flox^+/+^ Cre^–/–^ Lep^ob/+^ and GR Flox^+/+^ Cre^+/–^ Lep^ob/+^, which yielded GR Flox^+/+^ Cre^–/–^ Lep^ob/ob^ (*ob/ob* GR^fl/fl^), GR Flox^+/+^ Cre^+/–^ Lep^ob/ob^ (*ob/ob* GR-mKO), GR Flox^+/+^ Cre^–/–^ Lep^+/+^ (lean GR^fl/fl^), and GR Flox^+/+^ Cre^+/–^ Lep^+/+^ (lean GR-mKO).

An intraperitoneal injection of sodium pentobarbital (Kyoritsu Seiyaku) at 75 mg/kg body weight was used to anesthetize the mice. Blood samples were collected from the inferior vena cava, and blood glucose levels were measured using a Glucose Pilot Blood Glucose Monitoring System (NGP-01B, Technicon International). The remaining blood samples were transferred into heparinized sampling tubes, and plasma samples were separated by centrifugation. All plasma samples were kept at –80°C until biochemical assays were performed. Excised tissues were weighed and quickly frozen or fixed in formalin. Frozen tissues were crushed using Cryo-Press (Microtec Co., Ltd.) with a mini compressor (Kiso Power Tool Co.). For the IPITT and IPGTT, mice were fasted with free access to water and intraperitoneally injected with insulin (Humulin R, Eli Lilly) or glucose (FUJIFILM Wako Pure Chemicals Corporation). Fasting time, the dose of insulin or glucose, and time points for the measurement of blood glucose levels in tail vein are indicated in each legend.

### CT.

CT scans were performed using an in vivo micro X-ray CT system CosmoScan FX (Rigaku). The tube voltage was set at 90 kV and the current was constant at 88 μA. Mice were anesthetized with isoflurane (DS Pharma Animal Health) inhalation and scanned at a resolution of 120 μm pixels. The CT attenuation value of air was defined as –1000 Hounsfield units (HU) and that of water was defined as 0 HU. CT attenuation values for scanned images were calibrated using imaging phantoms for every scan.

### Histopathological analysis.

Immunohistochemistry was performed to measure fiber type–specific CSAs in gastrocnemius muscle. Excised muscle samples were stood upright on a piece of cork using tragacanth gum and frozen in liquid nitrogen–cooled isopentane. They were cut into 8-μm sections using a cryostat (Leica, CM1950) at –25°C. Cryosections were fixed in cold acetone (Nacalai Tesque Inc.) for 15 minutes and dried for 1 hour. After blocking with 5% goat serum/1% bovine serum albumin (BSA) in PBS, they were incubated overnight at 4°C with primary antibodies diluted in 1% BSA in PBS. Specimens were then washed with PBS and incubated with secondary antibodies diluted in 1% BSA in PBS at room temperature for 1 hour. After washing with PBS, they were mounted in Mount-Quick aqueous mounting medium (Daido Sangyo Co., Ltd.). Primary antibodies used for staining were as follows: rat IgG1 laminin α2 antibody (1:1,000, 4H8-2, Enzo Life Sciences), mouse IgG2b MyHC type I antibody (1:400, BA-D5), mouse IgG1 MyHC type IIa antibody (1:400, SC-71), and mouse IgM MyHC type IIb antibody (1:50, BF-F3). All MyHC antibodies were obtained from the Developmental Studies Hybridoma Bank. Secondary antibodies used for staining were as follows: an antibody labeled with Alexa Fluor 488 (1:1,000, A-11006, Thermo Fisher Scientific) for laminin α2 staining, and antibodies labeled with Alexa Fluor 568 (1:1,000, A-21144, A-21124, and A-21043, Thermo Fisher Scientific) for MyHC staining. Images of immunofluorescent staining were obtained using a BZ-X710 microscope system (KEYENCE). Fiber type–specific CSAs were quantified using ImageJ (64).

For histological analyses of WAT and liver, formalin-fixed tissues from each animal were cut into paraffin sections (5 μm thick) and subjected to hematoxylin and eosin (H&E) staining as previously described (65). Blinded observers measured CSAs of adipocytes using randomly selected cells and quantified manual outlining of the cellular membrane using ImageJ. The number of cells counted are indicated in each figure legend.

### Biochemical analyses of metabolic parameters.

Plasma levels of insulin, FGF21, and leptin were measured by ELISA (MS303, Morinaga Institute of Biological Science; MF2100, R&D Systems; MOB00B, R&D Systems, respectively) using an iMark Microplate Absorbance Reader (Bio-Rad). ALT activity in plasma was measured by colorimetric assay (K752-100, BioVision) using an iMark Microplate Absorbance Reader. These assays were accurately examined according to the manufacturer’s instructions. HOMA-IR was calculated using the formula HOMA-IR = blood glucose (mg/dL) × plasma insulin (μU/mL)/405 (66).

### Measurement of plasma amino acids.

Pooled plasma samples from 3 mice of each group were prepared, and metabolome analysis was performed using the Basic Scan package from Human Metabolome Technologies using capillary electrophoresis time-of-flight mass spectrometry (CE-TOFMS) based on methods described previously (67, 68). Peaks were extracted using the automatic integration software MasterHands (Keio University, Tsuruoka, Yamagata, Japan) to obtain peak information including *m/z*, peak area, and migration time.

### RNA-seq.

Total RNA was extracted from crushed tissues or cell pellets using Sepasol-RNA I Super G (Nacalai Tesque Inc.). Pooled RNA samples of tissue from 3 mice of each group were subjected to RNA-seq and analyzed as described previously (69), with slight modifications. Briefly, we used cutoff values of OD 260/280 ≥ 1.8 and RIN ≥ 7.0 for RNA quality, which are the recommended parameters of the Beijing Genomics Institute (BGI). Library construction and sequencing were performed on a DNBSEQ-G400 sequencer, and 100-bp paired-end reads were generated by BGI. After data filtering, mapping of reads and quantification were performed using Salmon v1.2.1 (https://salmon.readthedocs.io/en/latest/salmon.html). A fasta file listing protein-coding transcript sequences was downloaded from GENCODE (https://www.gencodegenes.org/mouse/release_M20.html) to be used as a reference. The output files were then imported to R v3.5.0 (https://www.r-project.org/) using the tximport package, and the countsFromAbundance parameter of the summarizeToGene function was set to “lengthScaledTPM.” Ensembl transcript IDs were converted to official gene names using biomaRt v2.38.0 (http://www.bioconductor.org/packages/release/bioc/html/biomaRt.html). Duplicated gene names were removed, and the entry with the largest total counts for each sample was retained. For analyses of differentially expressed genes using NOISeq (70), only genes with counts greater than 0 in all samples were included. DEGs were explored using the default settings (pnr = 0.2, nss = 5, v = 0.02, lc = 0), and a threshold of *q* = 0.9. GO enrichment analyses were performed using DAVID (https://david.ncifcrf.gov). The supercomputing resource was provided by the Human Genome Center, the Institute of Medical Science, the University of Tokyo (http://sc.hgc.jp/shirokane.html). Data generated were deposited in the NCBI Gene Expression Omnibus under accession numbers GSE198535 and GSE198536.

### Quantitative reverse transcription PCR.

Total RNA was subjected to reverse transcription with oligo-dT primer (Invitrogen) using a SuperScript III First-Strand Synthesis System for RT-PCR (Invitrogen). PCR was performed using a THUNDERBIRD Probe qPCR Mix or SYBR qPCR Mix (TOYOBO), Universal ProbeLibrary Sets (Roche), and a CFX96 Real-Time PCR Detection System (Bio-Rad). mRNA expression levels were calculated based on standard curves generated for each primer pair, and *36B4* mRNA was used as an invariant control. The sequences of primers used for quantitative reverse transcription PCR (qRT-PCR) are as follows: *36B4* forward 5′-ACTGGTCTAGGACCCGAGAAG-3′, reverse 5′-CTCCCACCTTGTCTCCAGTC-3′; *Fkbp5* forward 5′-AAACGAAGGAGCAACGGTAA-3′, reverse 5′-TCAAATGTCCTTCCACCACA-3′; *Foxo3* forward 5′-GCTAAGCAGGCCTCATCTCA-3′, reverse 5′-TTCCGTCAGTTTGAGGGTCT-3′; *MuRF1* forward 5′-CCTGCAGAGTGACCAAGGA-3′, reverse 5′-GGCGTAGAGGGTGTCAAACT-3′; *Atrogin1* forward 5′-AGTGAGGACCGGCTACTGTG-3′, reverse 5′-GATCAAACGCTTGCGAATCT-3′; *Tnf* forward 5′-CTGTAGCCCACGTCGTAGC-3′, reverse 5′-TTGAGATCCATGCCGTTG-3′; *F4/80* forward 5′-TCTCTAAACTCAAGGACACGAGGT-3′, reverse 5′-CTGGAAAATGCCCAGCAC-3′; and *GR* forward 5′-TGACGTGTGGAAGCTGTAAAGT-3′, reverse 5′-CATTTCTTCCAGCACAAAGGT-3′. GR primers were designed to span exons 3 and 4.

### Western blotting.

The tissues were solubilized using RIPA buffer containing 0.1% SDS, a phosphatase inhibitor cocktail, 100 nM MG-132, and 1 mM dithiothreitol. All reagents were purchased from Nacalai Tesque. Equal amounts of protein were then resolved by SDS-PAGE. Proteins were transferred to Immobilon (Millipore), blocked with 1% BSA, and then reacted with primary antibodies detecting GR (1:4000; 24050-1-AP, Proteintech), GAPDH (1:50,000; G9545, Sigma-Aldrich), or β-actin (1:1000; sc-47778, Santa Cruz Biotechnology). They were then reacted with HRP-labeled secondary antibodies (1:5000; NA934V and NA931V, GE Healthcare). Images were visualized using an Immobilon Western Chemiluminescent HRP Substrate (Millipore) and an ImageQuant LAS 4000 mini digital imaging system (GE Healthcare). Band intensities were analyzed using ImageJ.

### Cell culture.

C2C12 mouse myoblasts were obtained from the American Type Culture Collection and maintained in Dulbecco’s Modified Eagle Medium (DMEM) supplemented with 10% fetal calf serum (FCS) (Invitrogen). For experiments involving DEX stimulation after preincubation with insulin, C2C12 myoblasts were first spread and cultured in DMEM supplemented with 10% FCS for 24 hours. Then, the medium was changed to serum-free DMEM and the cells were incubated for 24 hours. The cells were then pretreated with human insulin (Nacalai Tesque Inc.) by adding it to the culture medium at the indicated concentration (0–50 nM). One hour later, 10 nM DEX was added to the culture medium, and 3 hours later, the cells were washed with PBS and harvested.

### Statistics.

For multiple-group comparisons, data were analyzed using 1- or 2-way ANOVA with Tukey-Kramer post hoc test. Exceptions were as follows: a 1-way ANOVA with Dunnett’s post hoc test was performed when 1 group was compared with the others; a 2- or 3-way repeated-measures ANOVA was performed to compare the results of IPGTT or IPITT. For 2-group comparisons, data were analyzed using 2-tailed Welch’s *t* tests, but when normality could not be assumed after evaluation using the Shapiro-Wilk test, data were analyzed using Mann-Whitney *U* tests. Right-skewed data were log-transformed before statistical tests as indicated in legends. Pearson’s product-moment correlation analyses were used to investigate correlation coefficients among parameters. *P* values below 0.05 were considered statistically significant. Bar graphs indicate mean ± SEM and dots indicate individual data points. In box-and-whisker plots, whiskers show the minimum and maximum values of the data set, the box shows 1 SD above and below the mean, and a line inside the box shows the median value. Statistical analyses were performed using GraphPad Prism 9. Graphs were made using GraphPad Prism 9, R, or Microsoft Excel.

### Study approval.

All animal experiments were performed with the approval of the Animal Ethics Committee of the Institute of Medical Science, the University of Tokyo (approval numbers, PA13-64, PH14-34, PH15-16, PH15-24, PH16-11, PH16-13, and PH16-26, PA18-26), and the approval of the Experimental Animal Committee of Keio University School of Medicine (approval number, 08024).

## Author contributions

HY, MU, NY, and HT designed the research project. HY, MU, NY, AKS, and YH conducted the experiments. HY, MU, AKS, NY, and HT analyzed the data. HY, MU, YK, and HT wrote the manuscript. HY, MU, NY, AKS, MY, YH, YK, MS, and HT read the manuscript critically.

## Supplementary Material

Supplemental data

Supplemental tables 1-12

## Version 1 03/14/2023

In-Press Preview

## Version 2 04/24/2023

Electronic publication
