# Supplementary material for: The crucial role of muscle glucocorticoid signaling in accelerating obesity and glucose intolerance via hyperinsulinemia
Source: JCI Insight. 2023 Apr 24;8(8):e162382. doi: 10.1172/jci.insight.162382 (PMC10243827; doi:10.1172/jci.insight.162382)
Supplement: Supplemental data [file jciinsight-8-162382-s054.pdf]

Supplementary Figure 1.

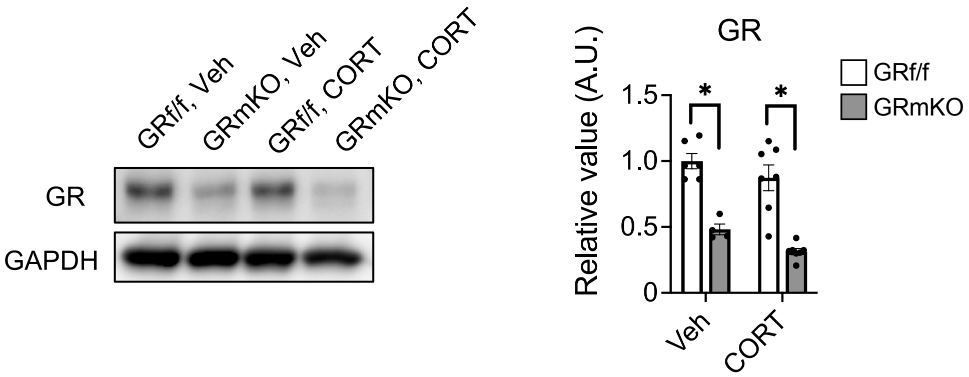

Supplementary Figure 1.
The expression of GR protein. Western blotting was performed using gastrocnemius muscle of GRf/f or GRmKO male mice treated with vehicle (Veh) or 100 μg/mL corticosterone (CORT) for 4 weeks. The band intensity of GR relative to GAPDH was quantified.

n = 4–7. Error bars represent mean ± SEM. *p < 0.05 determined by two-way ANOVA with Tukey–Kramer post hoc tests.

Supplementary Figure 2.

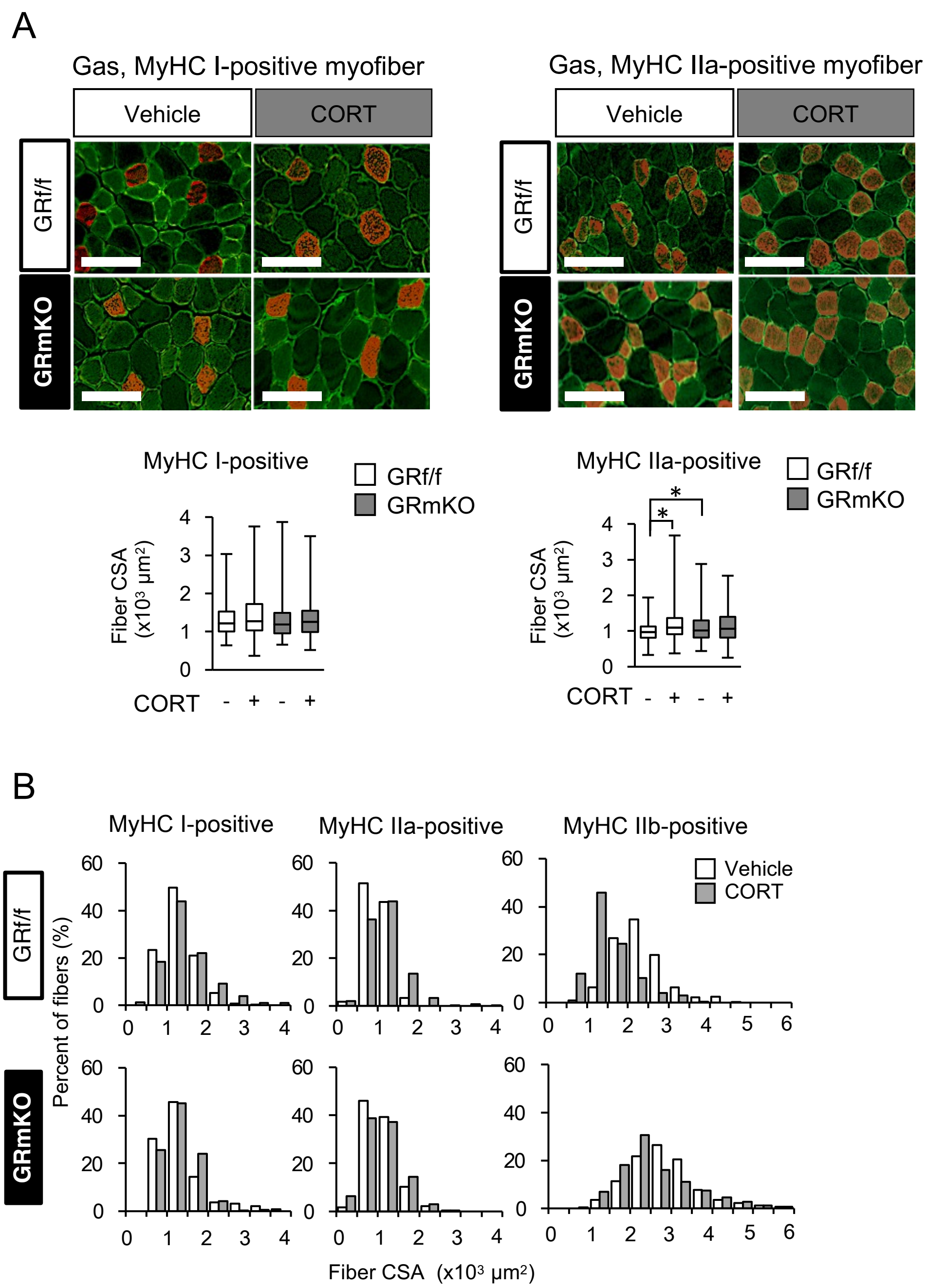

Supplementary Figure 2.
(A) Images of immunostaining for type I or IIa myosin heavy chain (red) and laminin (green) using transverse cryosections of gastrocnemius muscle (Gas) from GRf/f and GRmKO male mice treated with vehicle or CORT for 4 weeks. Scale bars represent 100 μm. Cross-sectional areas (CSAs) of each MyHC-positive myofiber are shown as boxplots. Whiskers show the minimum and maximum values of the dataset, the box shows one standard deviation above and below the mean, and a line inside the box shows the median value. A hundred fibers from each animal (n = 3–4) were counted. Statistical differences were determined by two-way ANOVA with Tukey–Kramer post hoc tests. *$p < 0.05$. (B) Frequency histograms of CSAs of MyHC I, IIa, and IIb-positive myofibers in GRf/f and GRmKO male mice treated with vehicle or CORT for 4 weeks.

Supplementary Figure 3.

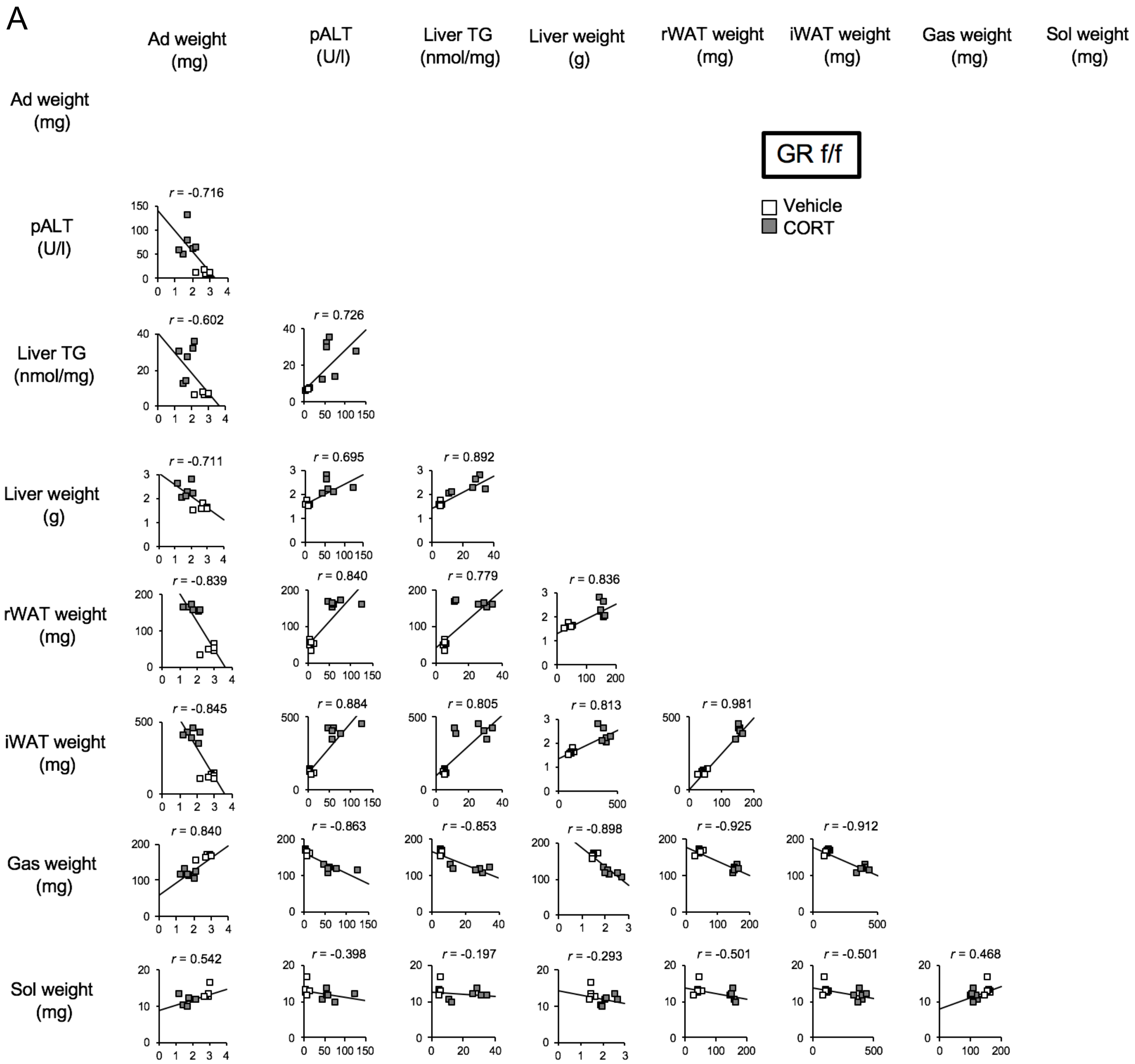

Supplementary Figure 3. *continued*

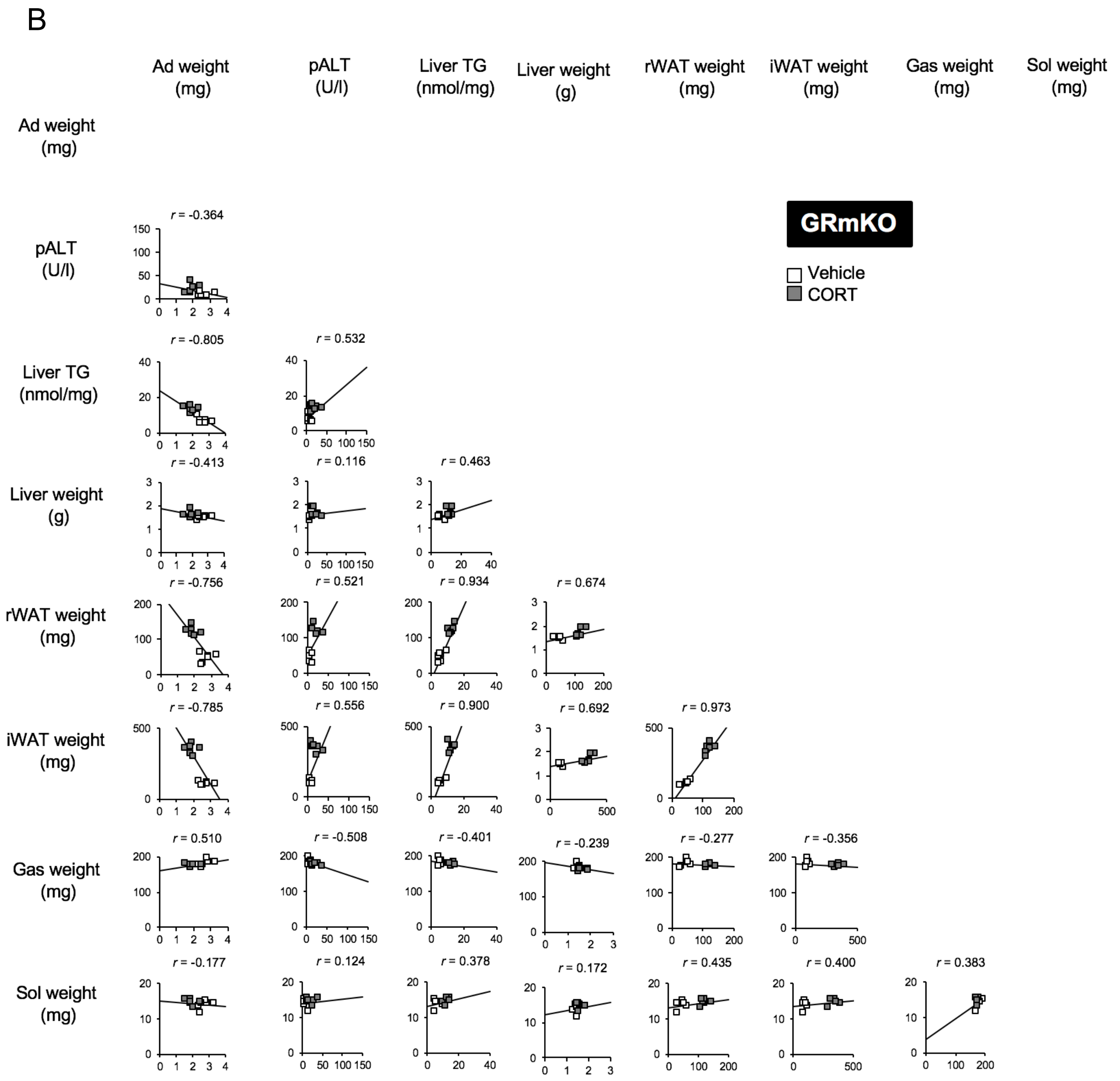

Supplementary Figure 3.
Scatter plots among each parameter of mice treated with vehicle (Vehicle, white squares) or corticosterone solution (CORT, gray squares) for 4 weeks.
(A) GRf/f mice. All graphs correspond to each cell in Figure 3A.
(B) GRmKO mice. All graphs correspond to each cell in Figure 3B.

Supplementary Figure 4.

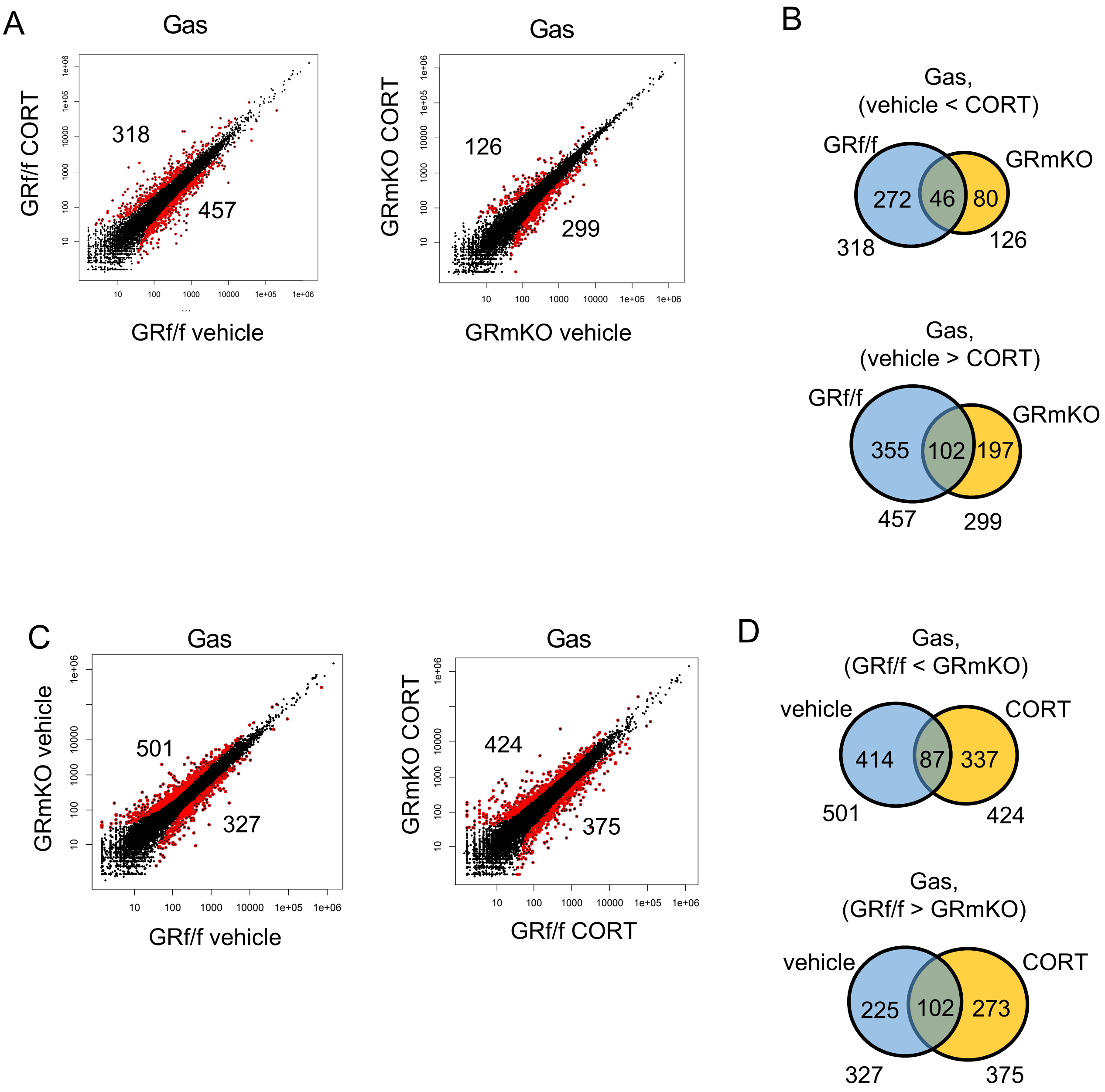

Supplementary Figure 4.

RNA sequencing (RNA-seq) was performed using pooled RNA samples (n = 3) of Gas from GRf/f and GRmKO mice treated with vehicle or CORT for 4 weeks. Differentially expressed genes (DEGs) were determined by NOISeq. In A and C, red dots represent DEGs, and numbers are their counts. Vertical and horizontal axes designate expected counts normalized by each library size and gene length. In B and D, values are the counts of DEGs.

(A, B) Genes increased or decreased by 4-week corticosterone (GRf/f vehicle vs. GRf/f CORT; GRmKO vehicle vs. GRmKO CORT) in Gas.

(C, D) Genes increased or decreased by muscle GR deletion (GRf/f vehicle vs. GRmKO vehicle; GRf/f CORT vs. GRmKO CORT) in Gas.

Supplementary Figure 5.

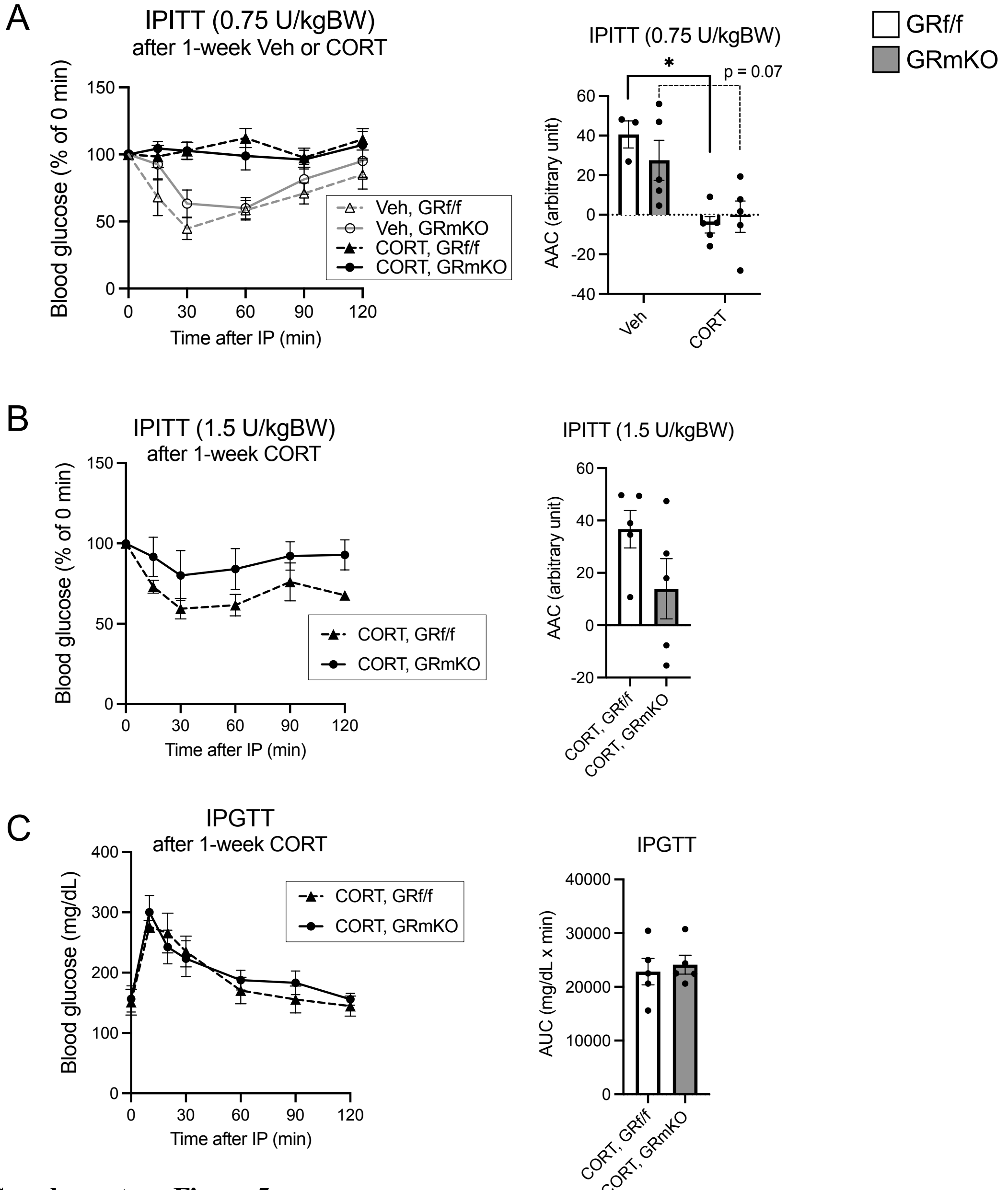

Supplementary Figure 5.

(A) The result of intraperitoneal insulin tolerance test (IPITT) after 1-week administration of CORT or vehicle (Veh). The mice after 4-hour fasting were intraperitoneally injected with 0.75 U/kg BW insulin. Blood glucose levels at 0, 15, 30, 60, 90, and 120 min were measured. Calculated area above the curve (AAC) is shown in the right panels.

(B) The result of IPITT after 1-week administration of CORT. The mice after 4-hour fasting were intraperitoneally injected with 1.5 U/kg BW insulin. Blood glucose levels at 0, 15, 30, 60, 90, and 120 min were measured. AAC is shown in the right panels.

(C) The result of intraperitoneal glucose tolerance test (IPGTT) after 1-week administration of CORT. The mice after 4-hour fasting were intraperitoneally injected with 1.5 g/kg BW glucose. Blood glucose levels at 0, 10, 20, 30, 60, 90, and 120 min were measured. Calculated area under the curve (AUC) is shown in the right panel.

Error bars represent mean ± SEM. Statistical analyses were conducted as follows (*$p < 0.05$).

In the left panel of (A), a 3-way repeated measures ANOVA was performed; $p < 0.001$ (time), $p < 0.001$ (CORT), $p = 0.475$ (genotype), $p < 0.001$ (time x CORT), $p = 0.325$ (time x CORT), $p = 0.291$ (CORT x genotype), $p = 0.931$ (time x CORT x genotype); Veh GRf/f (n = 3), Veh GRmKO (n = 5), CORT GRf/f (n = 5), and CORT GRmKO (n = 5). In the right panel of (A), a 2-way ANOVA with Tukey–Kramer post hoc tests was performed.

In the left panel of (B), a 2-way repeated measures ANOVA was performed; $p = 0.012$ (time), $p = 0.107$ (genotype), $p = 0.473$ (time x genotype). In the right panel of (B), Welch's t-test was performed. CORT GRf/f (n = 5), and CORT GRmKO (n = 5).

In the left panel of (C), a two-way repeated measures ANOVA was performed; $p < 0.001$ (time), $p = 0.778$ (genotype), $p < 0.667$ (time x genotype). In the right panel of (C), Welch's t-test was performed. CORT GRf/f (n = 5), and CORT GRmKO (n = 5).

Supplementary Figure 6.

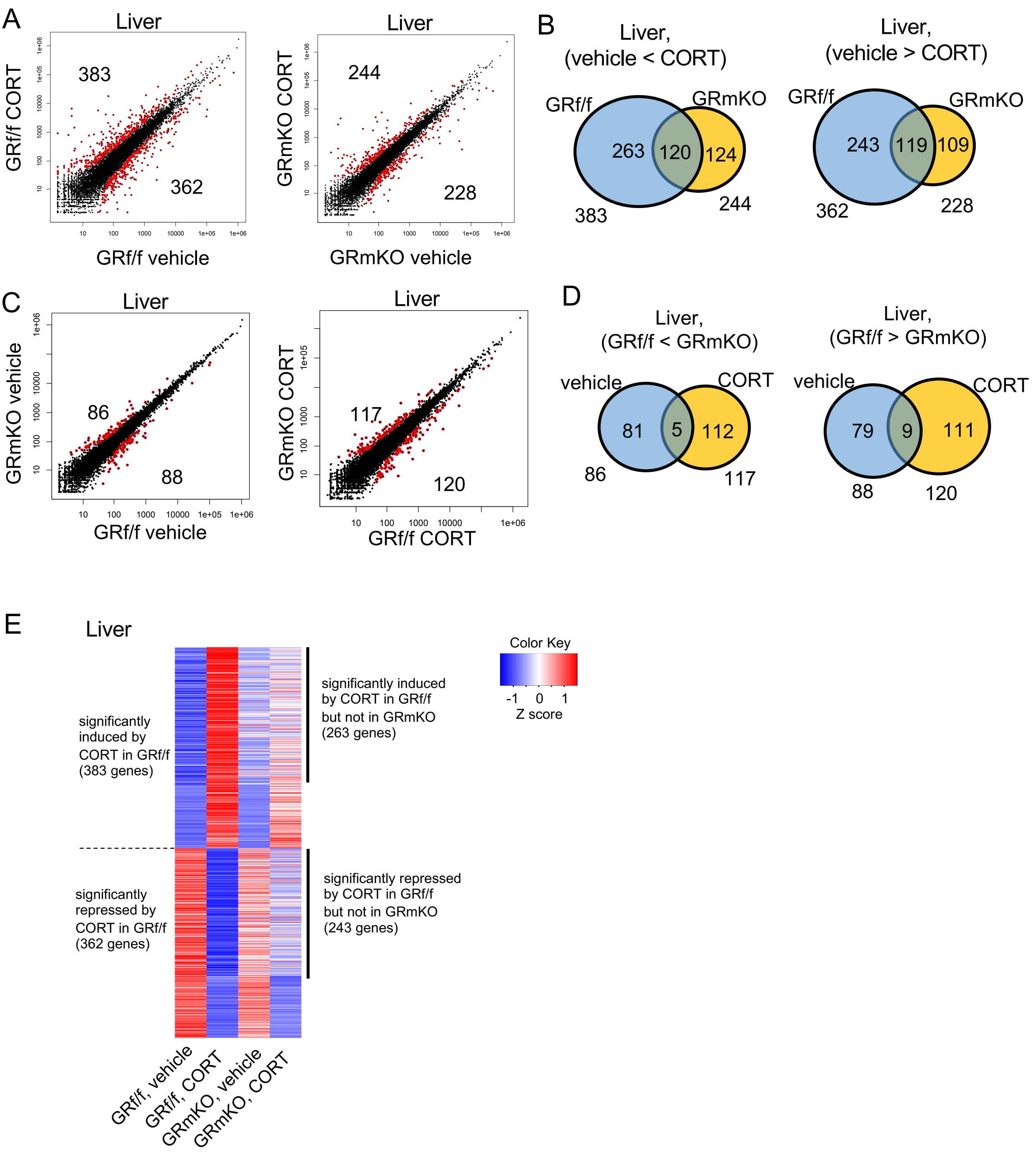

Supplementary Figure 6.
RNA sequencing (RNA-seq) was performed using pooled RNA samples (n = 3) of liver from GRf/f and GRmKO mice treated with vehicle or CORT for 4 weeks. Differentially expressed genes (DEGs) were determined by NOISeq. In A and C, red dots represent DEGs, and numbers are their counts. Vertical and horizontal axes designate expected counts normalized by each library size and gene length. In B and D, values are the counts of DEGs.

(A, B) Genes increased or decreased by 4-week corticosterone (GRf/f vehicle vs. GRf/f CORT; GRmKO vehicle vs. GRmKO CORT) in liver.

(C, D) Genes increased or decreased by muscle GR deletion (GRf/f vehicle vs. GRmKO vehicle; GRf/f CORT vs. GRmKO CORT) in liver.

(E) The levels of significantly induced or repressed genes by CORT in GRf/f mice are shown as a heatmap.

Supplementary Figure 7.

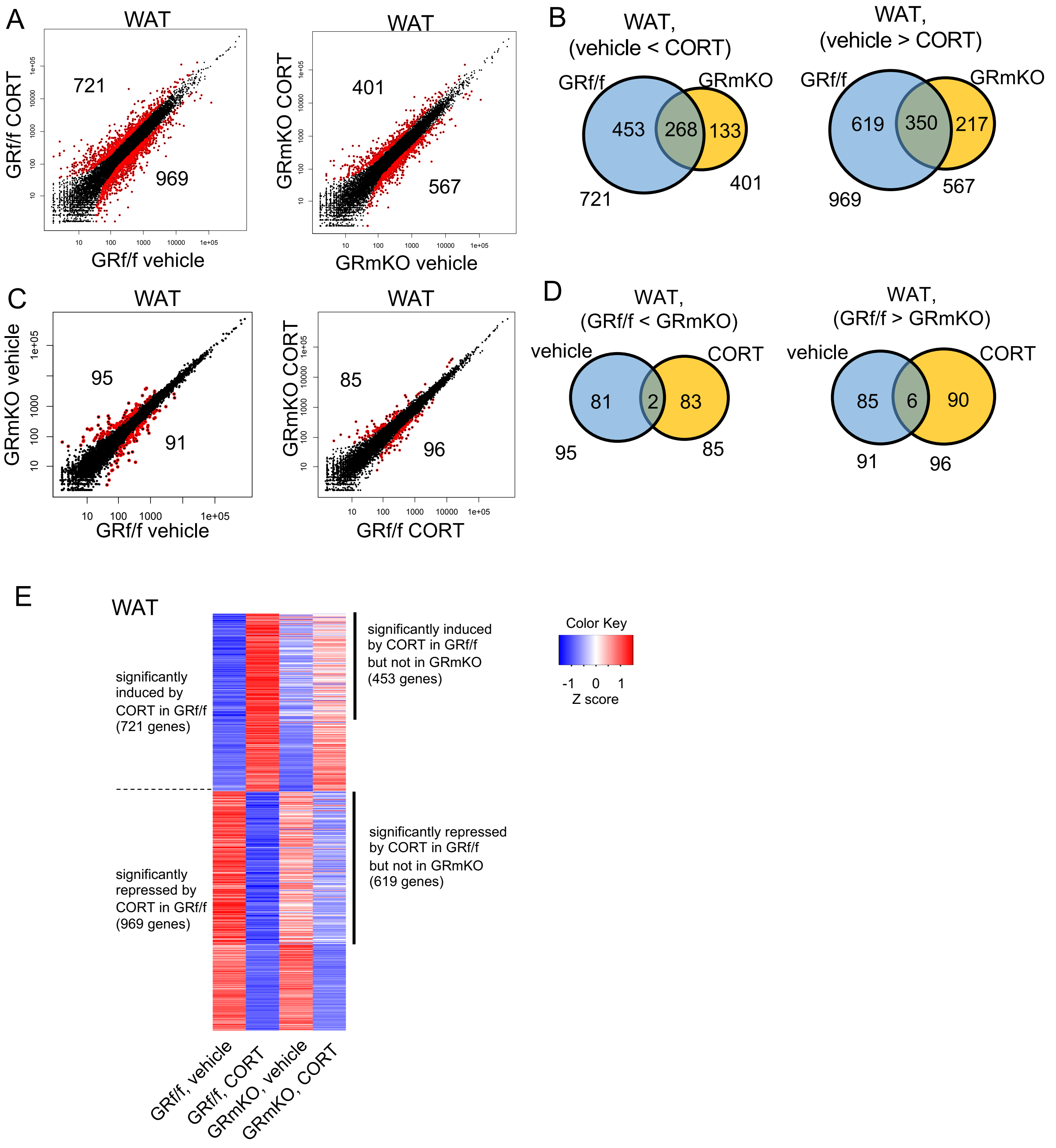

Supplementary Figure 7.

RNA sequencing (RNA-seq) was performed using pooled RNA samples (n = 3) of gonadal white adipose tissue (WAT) from GRf/f and GRmKO mice treated with vehicle or CORT for 4 weeks. Differentially expressed genes (DEGs) were determined by NOISeq. In A and C, red dots represent DEGs, and numbers are their counts. Vertical and horizontal axes designate expected counts normalized by each library size and gene length. In B and D, values are the counts of DEGs.

(A, B) Genes increased or decreased by 4-week corticosterone (GRf/f vehicle vs. GRf/f CORT; GRmKO vehicle vs. GRmKO CORT) in WAT.

(C, D) Genes increased or decreased by muscle GR deletion (GRf/f vehicle vs. GRmKO vehicle; GRf/f CORT vs. GRmKO CORT) in WAT.

(E) The levels of significantly induced or repressed genes by CORT in GRf/f mice are shown as a heatmap.

Supplementary Figure 8.

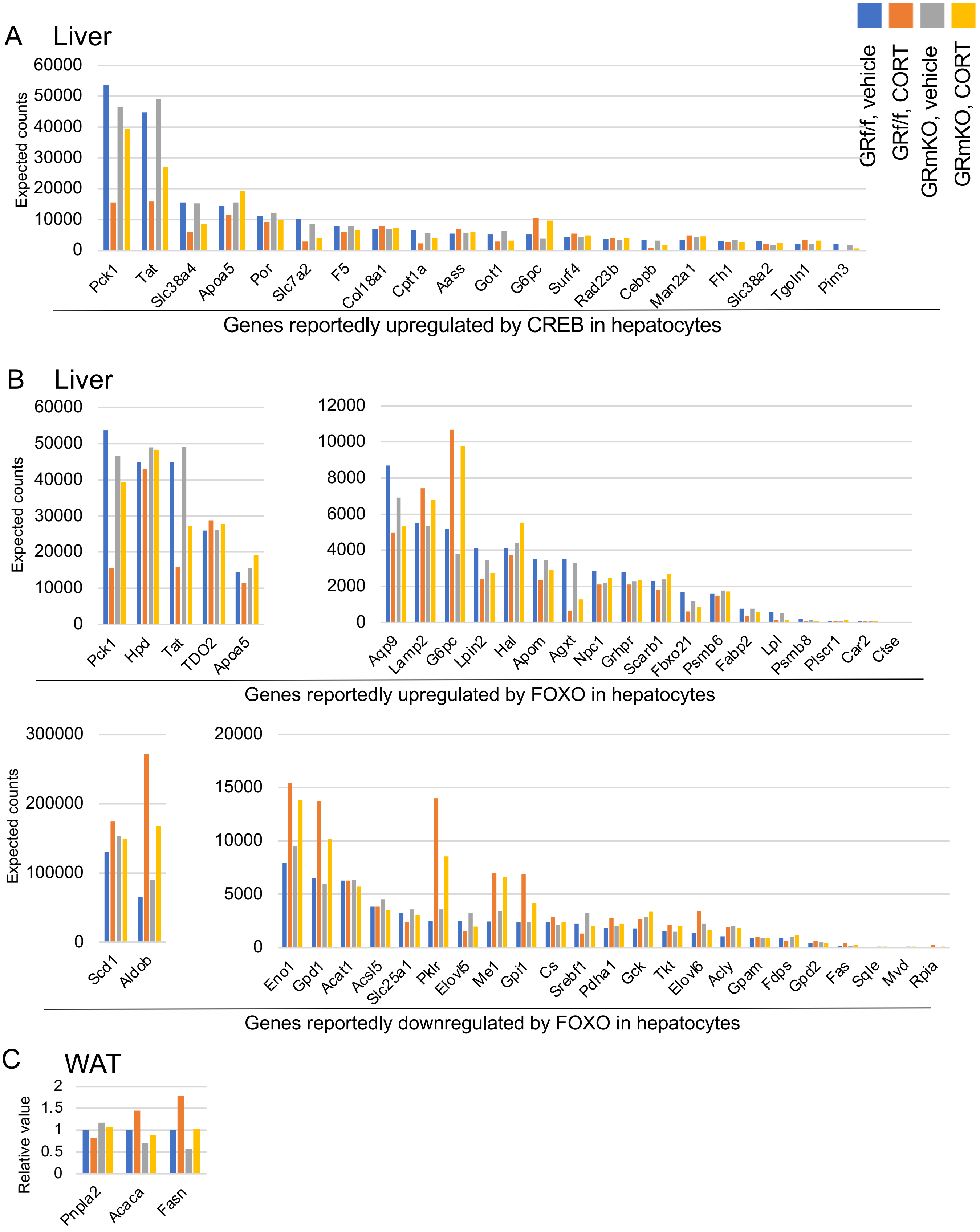

Supplementary Figure 8.

RNA sequence (RNA-seq) was performed using pooled RNA samples (n = 3) of each indicated tissue from GRf/f and GRmKO mice treated with vehicle or CORT for 4 weeks. Values are expected counts normalized by each library size and gene length. (A) Gene set which is reportedly regulated by CREB in hepatocytes (reference is described in the manuscript). Only the top 20 genes with high expression levels in the sample "GRf/f, vehicle" are shown. (B) Gene sets reportedly upregulated or downregulated by FOXO in hepatocytes (reference is described in the manuscript). (C) The expression of some genes related to lipid metabolism in gonadal white adipose tissue (WAT).

Supplementary Figure 9.

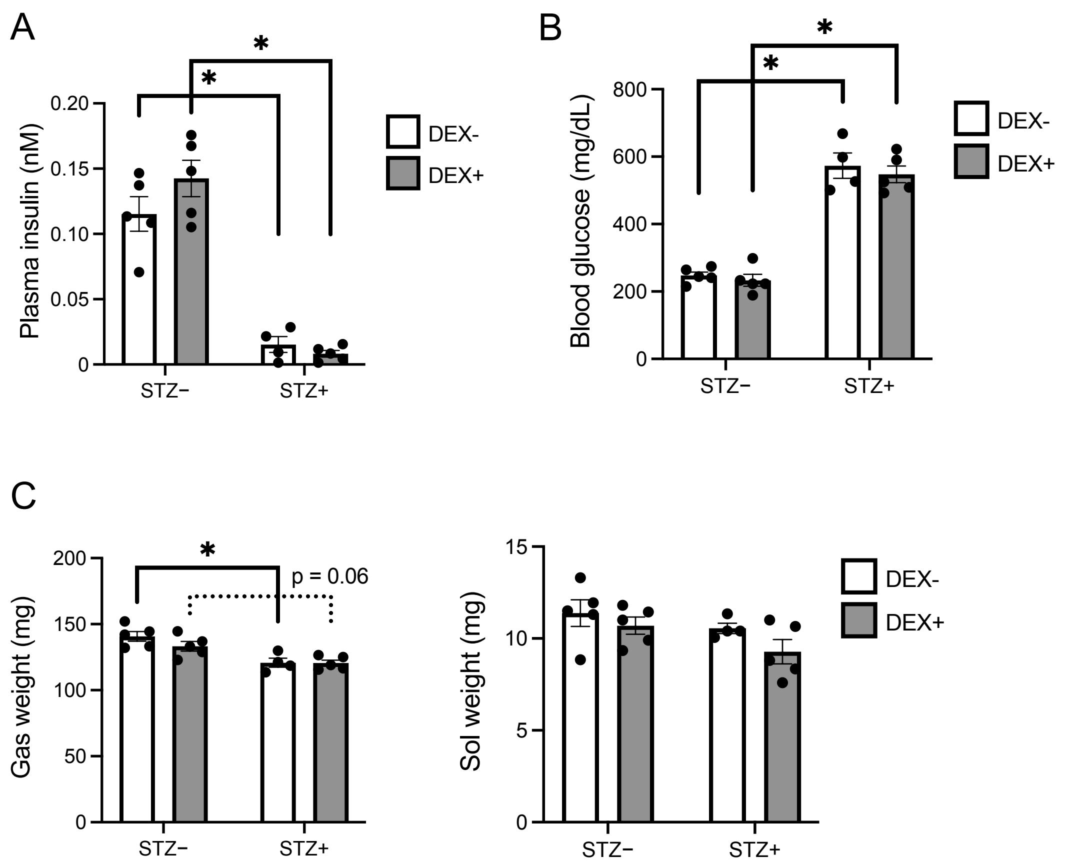

Supplementary Figure 9.

Seven-week-old male male mice were pretreated with vehicle (STZ−) or 200 mg/kg BW STZ (STZ+), followed by normal saline or 1 mg/kg BW DEX injection 5 days later. Plasma insulin levels (A), blood glucose levels (B), and the weight of Gas and Sol (C).

STZ, streptozotocin; DEX, dexamethasone; Gas, gastrocnemius muscle; Sol, soleus muscle.

n = 4–5. Error bars represent mean ± SEM. *$p < 0.05$ determined by two-way ANOVA with Tukey–Kramer post hoc tests.

Supplementary Figure 10.

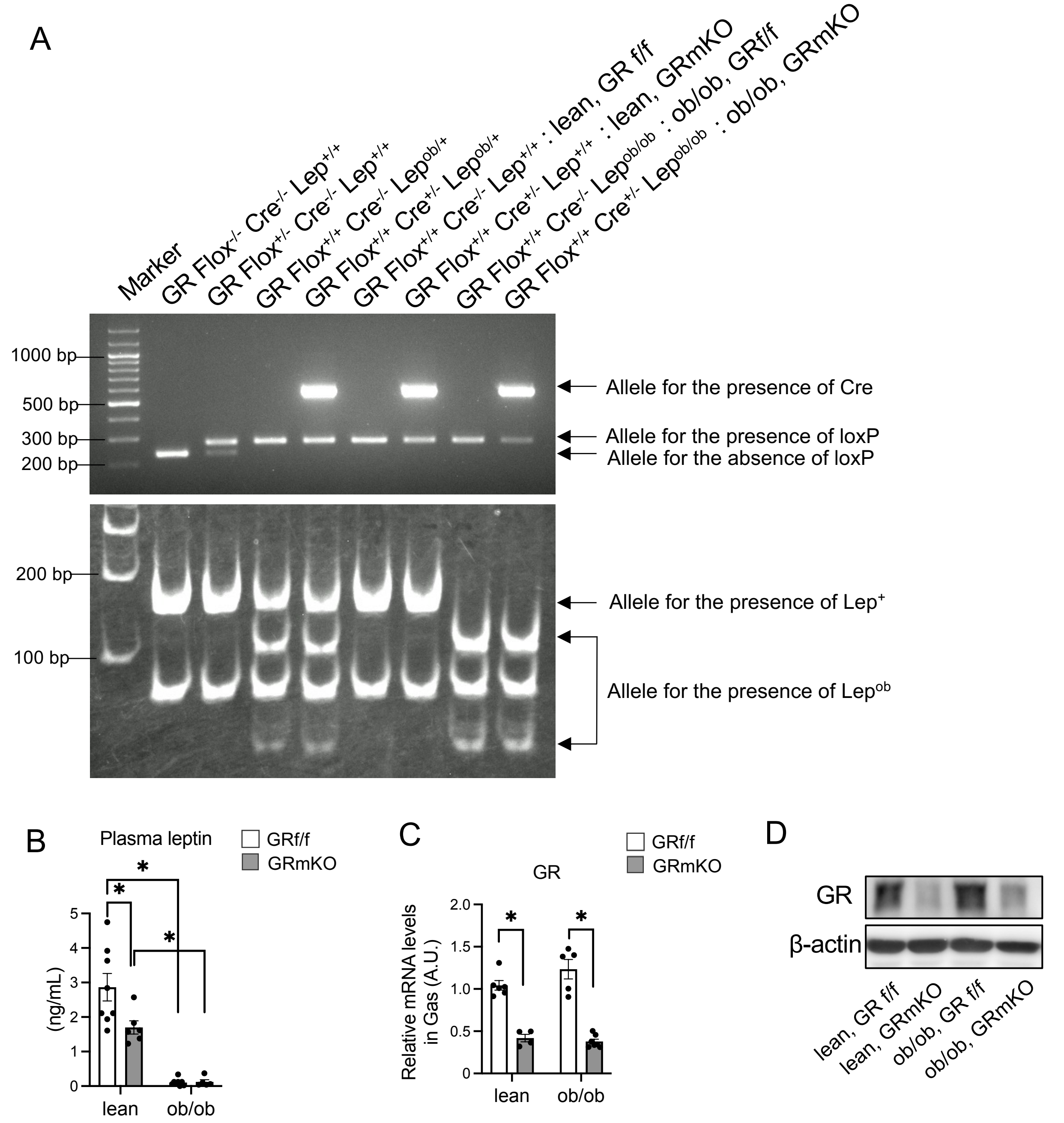

Supplementary Figure 10.

(A) Generation of skeletal muscle-specific GR knockout (KO) mice on ob/ob background. Mice were bred as described in Methods. PCR results leading to identification of lean GRf/f mice (GR Flox$^{+/+}$ Cre$^{-/-}$ Lep$^{wt/wt}$), lean GRmKO mice (GR Flox$^{+/+}$ Cre$^{+/-}$ Lep$^{wt/wt}$), ob/ob GRf/f mice (GR Flox$^{+/+}$ Cre$^{-/-}$ Lep$^{ob/ob}$), ob/ob GRmKO mice (GR Flox$^{+/+}$ Cre$^{+/-}$ Lep$^{ob/ob}$), and the other mice. (B) Plasma leptin levels of lean GRf/f, lean GRmKO, ob/ob GRf/f, and ob/ob GRmKO male mice (n = 5–8). Samples were collected in fed state. (C) GR (*Nr3c1*) mRNA levels in gastrocnemius muscle (Gas), normalized by the levels of 36B4 (*Rplp0*). qRT-PCR was performed. n = 4–7. (D) GR protein levels in Gas, assessed by western blotting.

Error bars represent mean ± SEM. *$p < 0.05$ determined by two-way ANOVA with Tukey–Kramer post hoc tests.

Supplementary Figure 11.

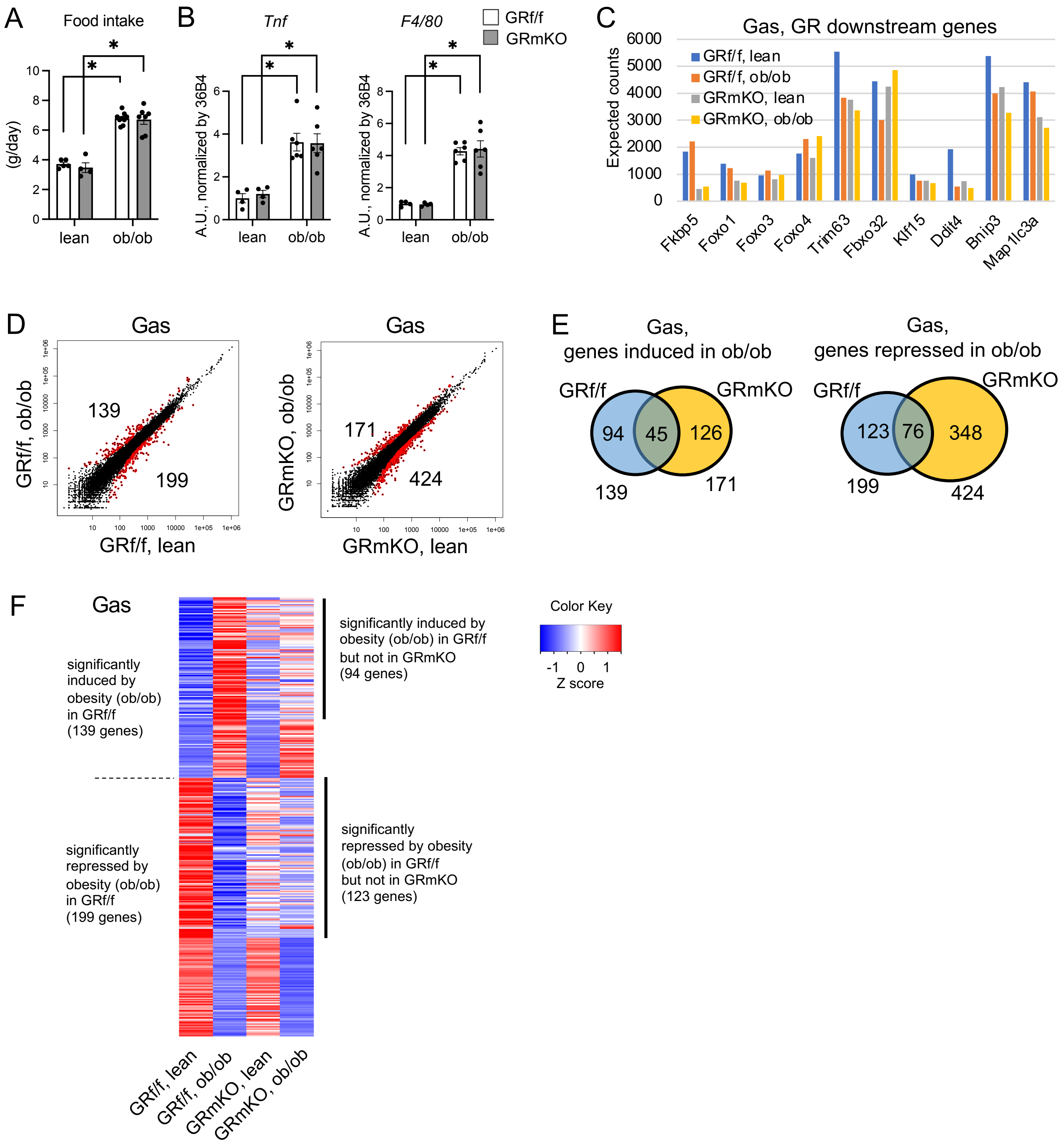

Supplementary Figure 11.
(A) Food intake of lean GRf/f, lean GRmKO, ob/ob GR f/f, and ob/ob GRmKO male mice. The weight of diet consumed during 6-week-old was measured (n = 4–9). (B) mRNA levels of gWAT of 7-week-old male mice in fed state assessed by qRT-PCR. Normalized by the levels of 36B4 (n = 4–6). (C-F) RNA sequence (RNA-seq) was performed using pooled RNA samples (n = 3) of Gas from 7-week-old male mice. The expected counts of some GR downstream genes (C). Differentially expressed genes (DEGs) determined by NOISeq, which induced or repressed by the effect of ob/ob background are shown in red dots with their counts (D). Venn diagrams of DEGs (E). Significantly induced or repressed genes by obesity (ob/ob) in GRf/f mice are shown as a heatmap (F). Error bars represent mean ± SEM. *$p < 0.05$ determined by two-way ANOVA with Tukey–Kramer post hoc tests.

Supplementary Figure 12.

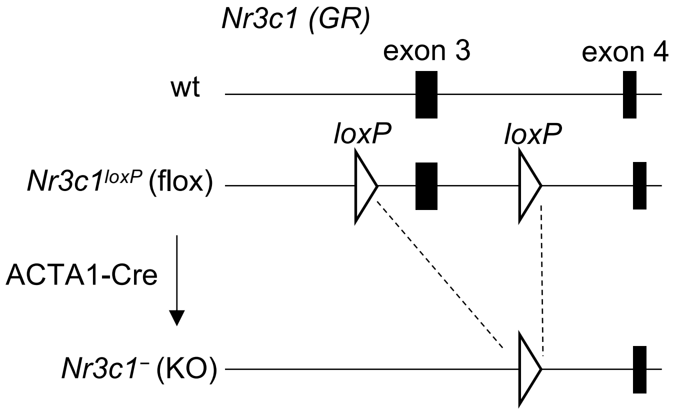

Supplementary Figure 12.
Diagram of the wild-type GR genomic locus (wt), the floxed GR allele (flox), and the GR allele obtained after Cre-mediated excision of exon 3 (KO).
